# CCL5 promotes the epithelial-mesenchymal transition of circulating tumor cells in renal cancer

**DOI:** 10.1186/s12967-024-05297-2

**Published:** 2024-09-03

**Authors:** Yibing Guan, Xueyi Liu, Juanhua Tian, Guang Yang, Fangshi Xu, Ni Guo, Lingyu Guo, Ziyan Wan, Zhixin Huang, Mei Gao, Tie Chong

**Affiliations:** 1 Department of Urology, School of Medicine, The Second Affiliated Hospital, Xi’an Jiaotong University, No 157 Xiwu Road, Xi’an, 710004 Shaan Xi China; 2 Department of Urology, The First Affiliated Hospital, Zhengzhou University, No 1 Jianshe East Road, Zhengzhou, 450052 He Nan China; 3 School of Chemistry, Xi’an Jiaotong University, Xi’an, 710049 Shaanxi China; 4 Henan Key Lab Reprod & Genet, The First Affiliated Hospital, Zhengzhou University, No 1 Jianshe East Road, Zhengzhou, 450052 He Nan China; 5 National & Local Joint Engineering Research Center of Biodiagnosis and Biotherapy, The Second Affiliated Hospital, School of Medicine, Xi’an Jiaotong University, No 157 Xiwu Road, Xi’an, 710004 Shaan Xi China

**Keywords:** CCL5, CTCs, Renal cancer, EMT, Prognosis

## Abstract

**Background:**

Circulating tumor cells (CTCs) are pivotal in tumor metastasis across cancers, yet their specific role in renal cancer remains unclear.

**Methods:**

This study investigated C–C motif chemokine ligand 5 (CCL5)'s tumorigenic impact on renal cancer cells and CTCs using bioinformatics, in vivo, and in vitro experiments. It also assessed renal cancer patients' CTCs prognostic value through Lasso regression and Kaplan–Meier survival curves.

**Results:**

Bioinformatics analysis revealed differential genes focusing on cellular adhesion and migration between CTCs and tumor cells. CCL5 exhibited high expression in various CTCs, correlating with poor prognosis in renal cancer. In 786-O-CTCs, CCL5 enhanced malignancy, while in renal cell carcinoma cell line CAKI-2 and 786-O, it promoted epithelial-mesenchymal transition (EMT) via smad2/3, influencing cellular characteristics. The nude mouse model suggested CCL5 increased CTCs and intensified EMT, enhancing lung metastasis. Clinical results shown varying prognostic values for different EMT-typed CTCs, with mesenchymal CTCs having the highest value.

**Conclusions:**

In summary, CCL5 promoted EMT in renal cancer cells and CTCs through smad2/3, enhancing the malignant phenotype and facilitating lung metastasis. Mesenchymal-type CTC-related factors can construct a risk model for renal cancer patients, allowing personalized treatment based on metastatic risk prediction.

**Supplementary Information:**

The online version contains supplementary material available at 10.1186/s12967-024-05297-2.

## Background

Renal cell carcinoma (RCC) accounts for approximately 2% of all cancer diagnoses and deaths worldwide, with approximately 295,000 new RCC cases diagnosed and approximately 134,000 related deaths occurring annually [1]. Despite the efficacy of surgical treatments, they can only temporarily slow disease progression or relieve symptoms, and 30% of patients with clear cell RCC still develop metastases [2]. At present, the follow-up examination of patients with RCC is primarily based on imaging. Unfortunately, there are no biological markers that can indicate possible early metastasis and recurrence before imaging diagnosis [3].

However, tumor circulating bodies in blood fluids, which are derived from tumors, can be used as markers either directly or indirectly. These bodies typically include circulating tumor deoxyribonucleic acid (DNA), circulating tumor cells (CTCs), extracellular vesicles, and tumor-induced platelets [4]. Specifically, CTCs-tumor cells shed from solid tumors into the blood-play a pivotal role in disease progression, particularly in tumor hematogenous metastasis, and are often considered the foundation of tumor metastasis [5]. Utilizing CTCs detection allows for the non-invasive dynamic monitoring of tumors. Furthermore, it ensures swift investigation of suspected metastasis and recurrence even before diagnoses stemming from abnormal imaging. Additionally, the detection outcomes can be leveraged for cancer diagnosis, screening, and prognosis prediction [6]. In 2017, the American Joint Committee on Cancer adopted CTCs as the M0 (i +) staging standard for breast cancer.

In our prior research, we observed that an elevated proportion of mesenchymal CTCs in RCC patients might be associated with tumor metastasis [7, 8]. In this study, our objective was to elucidate the role and mechanism of epithelial-mesenchymal transition (EMT) in RCC's CTCs. Our findings confirmed that the C–C motif chemokine ligand 5 (CCL5) can stimulate EMT and foster the malignant phenotypes of renal cancer cells through the smad pathway. This led to an increase in the number of CTCs, a decrease in the proportion of E-cadherin-positive CTCs, and an enhancement in the metastatic potential of tumor cells, particularly in the lungs. Concurrently, we implemented a straightforward and effective risk stratification model for renal cancer metastasis, heavily influenced by mesenchymal CTC-related factors.

## Methods

### Bioinformatics analysis

Statistical analyses and bioinformatics data visualization were conducted using R software. We extracted datasets for CTCs and the corresponding tumor cell series GSE67980, GSE111065, GSE74981, GSE144561, GSE82104, and GSE85610 (Table S1) from the Gene Expression Omnibus (GEO, https://www.ncbi.nlm.nih.gov/geo/) database. We employed the GEOquery package to refine and standardize the data. Following this, we utilized the limma package for differential gene analysis. Gene ontology (GO) and Kyoto Encyclopedia of Genes and Genomes (KEGG) analyses were conducted using the Database for Annotation, Visualization, and Integrated Discovery tool (https://david.ncifcrf.gov/). We used the ComplexHeatmap package to display genes with varying expression levels in the expression profile. Through the CytoHubba plug-in, we computed the radiality scores and pinpointed the top 10 hub genes boasting the highest scores (Table S2). The ctcRbase database aided us in visualizing the expression levels of CCL5 across diverse locations or organs in various cancer types. Lastly, we employed the OncoLnc tool to ascertain the prognostic significance of CCL5 in patients diagnosed with RCC.

### 786-O-CTC construction

786-O cells were infected with a green fluorescent protein-luciferase-puromycin (GFP-LUC-Puro) triple-labeled control lentivirus (HanBio Biotechnology Co, China) to generate a monoclonal stable cell line. Following cell expansion, the cells were administered into non-obese diabetic/severe combined immune deficiency mice via tail vein injection at a concentration of 3 × 10^6^/200 μL. The mice were then maintained under standard conditions in an specific-pathogen-free (SPF) environment for 30 days. During this period, small animal imaging confirmed the development of lung metastases in the mice. Blood samples from mice with lung metastases were extracted from the heart, leading to the isolation of CTCs via filtration with a cell strainer. Using tweezers in a sterile workspace, the filter membrane was detached from the strainer and positioned in a 60 mm cell culture dish with the cell-attachment surface exposed. Roswell Park Memorial Institute (RPMI) 1640 complete medium (Gibco Life Technologies, USA) with 2 μg/mL puromycin (Beyotime Biotechnology Co, China) was added to the dishes, and the medium swapped based on cellular growth while discarding non-viable floating cells (Fig. 2A). Leukocytes, either free-floating or attached, were eliminated either by medium exchange or puromycin. The 786-O-CTCs adhered and proliferated, with the subsequent expanded cells cryopreserved for future application. In experiments involving 786-O-CTC, maraviroc, a CCL5 receptor CCR5 inhibitor (Topscience Biotechnology Co, China), was utilized as an intervention, the working conditions were to incubate the cells for 24 h at a concentration of 10 nM. Ribonucleic acid (RNA) from cells was extracted using TRIzol reagent (Invitrogen, USA) following the manufacturer's guidance. Finally, the RNA samples underwent sequencing and assessment at Personal Biotechnology Co (China).

### Cell culture and transfection

The renal cancer cell lines, 786-O and CAKI-2, were sourced from Procell Biotechnology Co (China). The cell lines had been authenticated using short tandem repeat profiling. All experiments were performed with mycoplasma-free cells. Both 786-O and 786-O-CTC cells were cultured in RPMI1640 medium, supplemented with 10% fetal bovine serum (Biological Industries, Israel). In contrast, CAKI-2 cells thrived in McCoy’s 5A medium, also furnished by Procell Biotechnology Co, and enriched with 10% fetal bovine serum. All cells were housed in a humidified incubator, where they were exposed to 5% CO_2_ at 37 °C.

For experimentation, 786-O and CAKI-2 cells were planted in 6-well plates. When the cell density attained 40%, a 60 μL volume of lentivirus (boasting a titer of 10^8^ transduction units /mL) was introduced. Lentiviruses, designed for CCL5 overexpression, knockdown, and serving as negative controls, were synthesized by HanBio Biotechnology Co (China). Following a 2 week selection phase with 2 μg/mL puromycin procured from Beyotime Biotechnology Co (China), the efficiency of both knockdown and overexpression was authenticated through quantitative real-time polymerase chain reaction (qRT-PCR) and enzyme-linked immunosorbent assays (ELISA).

### Cell counting kit—8 (CCK8) assays

The CCK8 assays were employed to evaluate cell proliferation. Cells were plated in 96-well plates, with each well accommodating 3,000 cells. Subsequently, the plates were incubated at 37 °C in an environment of 5% CO_2_. Proliferation testing took place at intervals of 0 h, 24 h, 48 h, and 72 h. Post a 2 h incubation in a 10% CCK8 solution at 37 °C, cell proliferation metrics were derived by gauging the absorbance at 450 nm using a microplate reader from Thermo Corporation, USA. In the context of cellular phenotype and molecular experiments, the smad pathway in 786-O cells was modulated by employing the smad3 specific inhibitor (SIS3). This inhibitor, obtained from Topscience Biotechnology Co (China), was used at a 5 μM concentration for 24 h as part of a rescue experiment.

### ELISA assays

Centrifuged the cell culture medium for analysis at 300 g for 10 min to precipitate solids. Brought all reagents and samples to room temperature before use. According to the ELISA kit (MultiSciences Biotechnology Co, China) manual, diluted necessary reagents to their working concentrations, prepare a CCL5 standard curve using human standards, and cell culture supernatant samples. Filled each ELISA well with 300 μL wash buffer, soaked for 30 s, and then dried the plate. Added 100 μL of the standard or medium for blanks, and 100 μL of supernatant for samples to the designated wells. Introduced 50 μL of the detection antibody into each well. Sealed and incubated the plate at room temperature for 2 h. Discarded the contents and washed each well six times with 300 μL wash buffer. Added 100 μL of horseradish peroxidase (HRP)-conjugated streptavidin to each well, sealed, and incubated at room temperature for 45 min. After discarding the liquid and washing six times, added 100 μL of 3,3′,5,5′-tetramethylbenzidine substrate to each well, incubated for 20 min in the dark, and stopped the reaction with 100 μL of stop solution, changing the color from blue to yellow. Used an ELISA reader for dual-wavelength detection at 450 nm minus 630 nm. Calculated the mean optical density (OD) values for standards and samples, subtracting the zero standard OD, to plot the standard curve and establish the best fit.

### Cell cycle assays

After releasing adherent cells through trypsinization, they were immersed in 500 μL of pre-cooled ethanol (70% volume fraction) for fixation at 4 °C overnight. Next, according to the cell cycle assays kit (KeyGEN Biotechnology Co, China) manual, a solution combining RNaseA and propidium iodide (PI) was formulated at a 1:9 volume ratio. The fixative was carefully rinsed off using 1 × phosphate-buffered saline (PBS). The cells were then treated with 500 μL of the freshly prepared PI/RNaseA working solution and allowed to incubate at room temperature for 30 min, shielded from light. Using a flow cytometer (ACEA Biosciences, USA) with an excitation wavelength of 488 nm, red fluorescence emitted by cells was collected. The cell cycle-related results were then obtained through analysis using the accompanying NovoCyte software.

### Apoptosis assays

According to the apoptosis assays kit (BIOSCIENCE Biotechnology Co, China) manual, cells were collected using trypsinization, avoiding the use of ethylenediaminetetraacetic acid (EDTA), and subsequently resuspended in 1 × Annexin V binding buffer. Then, 100 μL of the cell suspension was transferred to a flow cytometry tube. To this mixture, 5 μL of Annexin V conjugated to phycoerythrin (PE) (known as Annexin V-PE) and 5 μL of Nuclear Red II were introduced. After ensuring thorough mixing, the cells were allowed to incubate at room temperature for 15 min in a light-shielded environment. Following this incubation, 400 μL of 1 × Annexin V binding buffer was incorporated, and the mixture was thoroughly combined, prepping it for flow cytometry analysis. For the flow cytometry, Annexin V-PE was excited with a laser at a 488 nm wavelength, and its fluorescence emission spectrum was captured at 578 nm using the NovoCyte flow cytometer from ACEA Biosciences, USA. In the case of Nuclear Red II, it was excited at a wavelength of 638 nm, with the FL4 channel designated for its detection. The cell apoptosis-related results were then obtained through analysis using the accompanying NovoCyte software.

### Transwell migration assays

Cells were resuspended in serum-free medium, and a concentration of 30,000 cells per well was introduced into the upper chamber of a 24-well transwell plate from Millipore, USA. The lower chamber received the complete medium. After a 24 h incubation, cells underwent fixation using anhydrous methanol for a duration of 15 min and were then subjected to staining with 0.1% crystal violet (Beyotime Biotechnology Co, China), also for 15 min. Following this, the cells from the upper surface were gently removed using a cotton swab, the chambers rinsed with PBS, and the migrated cells on the bottom surface were visualized and enumerated using an inverted microscope. The invasion assays mirrored the aforementioned procedure, with an additional preliminary step where the upper chamber was coated with Matrigel from BD Biosciences, USA. It's worth noting that all transwell migration and invasion experiments employed a chamber with a pore size of 8 μm.

### Colony formation assays

Cells were seeded into 6-well plates at a density of 1000 cells per well and were subsequently cultured in an incubator set at 37 °C with 5% CO_2_. After a 2 week period, these cells were fixed using 4% paraformaldehyde and then stained with crystal violet. Following staining, colonies with more than 30 cells were identified and counted to evaluate colony formation.

### Western blot analysis

After washing the cells twice with pre-cooled 1 × PBS, they were lysed on ice using radioimmunoprecipitation assay buffer, which contained 1 mM phenylmethylsulfonyl fluoride. To quantify the total protein concentration, a bicinchoninic acid assay kit from Beyotime Biotechnology (China) was employed. The protein samples underwent separation on a 10% sodium dodecyl sulfate–polyacrylamide gel and were then transferred to a polyvinylidene fluoride membrane (Millipore, USA). Following a block with 5% skim milk at approximately 26 °C for 1 h, the membrane was exposed overnight at 4 °C to a series of primary antibodies, including: anti-matrix metallopeptidase 9 (Proteintech, China), anti-smad2 (Proteintech, China), anti-E-cadherin (Immunoway, USA), anti-slug (Immunoway, USA), anti-vimentin (Immunoway, USA), anti-smad3 (CST, USA), anti-zinc finger protein SNAI1 (Abcam, USA), anti-phospho-smad3 (Abcam, USA), anti-phospho-smad2 (Immunoway, USA), anti-N-cadherin (Abcam, USA), and anti-glyceraldehyde 3-phosphate dehydrogenase (Proteintech, China). Subsequent to primary antibody incubation, the membrane strips were exposed to HRP-conjugated secondary antibodies (Immunoway, USA) for 1 h at ambient conditions. The antibodies were diluted in line with the recommended dilution ratios provided in the manual. In the concluding step, the blots underwent visualization using the ChemiDoc MP imaging system (Bio-Rad Corporation, USA) paired with an enhanced chemiluminescence kit (Millipore, USA).

### RNA extraction and RT-PCR analysis

In compliance with the manufacturer’s guidelines, TRIzol reagent (Invitrogen, USA) was utilized for the extraction of total cell RNA. Subsequently, reverse transcription was achieved using the PrimeScript™ RT Master Mix (TaKaRa Bio, Japan) kit. For qPCR, the SYBR® Premix Ex Taq™ I (TaKaRa Bio, Japan) reagent was employed on a BioRad CFX96 detection system. The primer sequences, which were custom-synthesized by Sangon Biotech (China), are provided below:

*CCL5*: Forward 5′—ACCAGTGGCAAGTGCTCCAAC—3′, Reverse 5′—CTCCCAAGCTAGGACAAGAGCAAG—3′

*GAPDH*: Forward 5′—GAGTCAACGGATTTGGTCGT-3′, Reverse 5′—GACAAGCTTCCCGTTCTCAG—3′

*N-cadherin*: Forward 5′—CCTCCAGAGTTTACTGCCATGAC—3′, Reverse 5′-GTAGGATCTCCGCCACTGATTC—3′

*E-cadherin*: Forward 5′—GCCTCCTGAAAAGAGAGTGGAAG—3′, Reverse 5′—TGGCAGTGTCTCTCCAAATCCG—3′

*Snail*: Forward 5′—TCTAGGCCCTGGCTGCTACAA—3′, Reverse 5′-ACATCTGAGTGGGTCTGGAGGTG—3′

*Slug*: Forward 5′—GAGCATTTGCAGACAGGTCA-3′, Reverse 5′—ACAGCAGCCAGATTCCTCAT—3′

*Vimentin*: Forward 5′—GTGAATCCAGATTAGTTTCCCTCA-3′, Reverse 5′-CAAGACCTGCTCAATGTTAAGATG-3′

*MMP-9*: Forward 5′—GCCACTACTGTGCCTTTGAGTC-3′, Reverse 5′—CCCTCAGAGAATCGCCAGTACT—3′.

After preparation of the reaction system, amplification was performed using a real-time quantitative PCR instrument (Thermo Fisher Scientific, USA). The results were analyzed using QuantStudio™ Design & Analysis SE Software, with transcription levels of different markers compared based on the 2^-ΔΔCt value.

### Animals and treatment

Male BALB/c-nu mice, aged 4–5 weeks, were procured from and subsequently raised in SPF conditions at the animal experiment center of Xi’an Jiaotong University. For the study, the mice were divided into two random groups, each group consisted of 3 mice. In the first group, lentivirus control (LV-Con) 786-O cells were injected, while the second group received lentivirus-CCL5 (LV-CCL5) 786-O cells. Both injections were made into the left armpits, with a cell count of 5 × 10^6^. Tumor dimensions, both length and width, were regularly measured every 7 days. Their volume was deduced using the formula: volume = length × width × width/2. After a period of 28 days, the mice underwent euthanasia via cervical dislocation. Subsequently, the tumors were removed and weighed. In a distinct experiment, two new random groups of mice were established, each group consisted of 5 mice. The first set received injections of LV-Con 786-O cells, and the second of LV-CCL5 786-O cells. This time, injections were administered via the tail vein, each containing 4 × 10^6^ cells. A span of 10 days post-injection, in vivo imaging was executed using equipment from Caliper Company, USA. During the imaging procedure, the mice were anesthetized using 30 mg/kg of pentobarbital. Following the imaging, blood samples were collected directly from the heart. All organs were harvested and fixed in a 4% paraformaldehyde solution, and then preserved in paraffin. The blood samples were processed through a cell strainer, aiming to isolate CTCs. These CTCs would later undergo immunofluorescence detection, specifically targeting the anti-E-cadherin antibody (Immunoway, USA). It's noteworthy that the in vivo imaging, blood collection, and the euthanasia process for organ procurement were carried out every 7 days. Every procedure involving the animals adhered strictly to the animal experimentation guidelines and received full approval from the institutional ethics committee at the second affiliated hospital of Xi’an Jiaotong University.

### Hematoxylin–eosin (H&E) staining assay

According to the H&E kit (Beyotime Biotechnology Co, China) manual, the 4 μm-thick tissue sections were first deparaffinized and subsequently rehydrated. Following this, the sections underwent hematoxylin staining for 5 min and were then counterstained with eosin for an additional 3 min. Subsequent to staining, the sections were methodically dehydrated using a graded alcohol series: 75%, 85%, 95%, and finally 100%. Upon completion, the stained sections were examined under a microscope.

### Immunofluorescence

The remaining sections underwent deparaffinization in xylol solvent and were rehydrated through a series of ethanol washes, followed by distilled water. They were then treated for antigen retrieval in sodium citrate buffer (pH 6.0) at 95 °C for 10 min. To prevent non-specific labeling, sections were pre-treated with an immunofluorescence blocking solution (Beyotime Biotechnology Co, China) prior to the primary antibody application. The sections were then incubated overnight in a 4℃ humidified chamber with anti-E-cadherin (ImmunoWay, USA). The following day, after washing with PBS, the sections were exposed to goat anti-rabbit IgG Coralite594 (Proteintech, China) and incubated at room temperature for an hour. The antibodies were diluted in line with the recommended dilution ratios provided in the manual. Subsequent to a final PBS rinse, cell nuclei were highlighted using 4,6-diamidino-2-phenylindole (DAPI). The stained sections were then mounted with coverslips and visualized under a fluorescence microscope.

### Patient clinical data

Under the approval of the ethics review committee of the Second Affiliated Hospital of Xi’an Jiaotong University, we enrolled 117 RCC patients who underwent surgery at our institution between 2015 and 2021. The detailed inclusion and exclusion criteria for patient selection were illustrated in Figure S6. The institutional review board sanctioned the utilization of clinical samples, and every participant provided their written informed consent. All research methodologies adhered to the Declaration of Helsinki guidelines. The patients' clinical data encompassed factors such as sex, age, tumor stage, and pathological type, among others. Updates on the patients' progress were sourced from our hospital’s follow-up system database. Post-surgery, within a span of 3–12 months, patient blood samples were collected for CTCs testing. It is noteworthy that none of these patients received steroids or anti-inflammatory medications that might influence blood immune cell counts prior to this collection.

### CTCs capture and typing

Following the standardized protocol for CTCs testing, peripheral blood samples (5 ml, anticoagulated with EDTA) were drawn from each participant. Erythrocyte lysis buffer (Sigma, USA) was introduced to isolate mononuclear cells. Subsequently, the CanPatrol CTCs enrichment technique (SurExam Bio-Tech Co., China) was employed for CTCs separation. These blood samples were channeled from the storage tube to the filter, retaining the mononuclear cells atop the filter's nanomembrane. Using RNA-fluorescence in situ hybridization, the fixed cells underwent typing to categorize the CTCs types. This CTCs typing procedure harnessed various RNA probes targeting epithelial type-specific genes, namely *EpCAM*, *CK8*, *CK18*, and *CK19*, and mesenchymal-specific genes, including *vimentin* and *twist* (refer to Table S5 for the probe sequence). The amplification probe synchronized with the aforementioned labeling probe, acquiring fluorescent group labels to generate fluorescence signals. Based on morphological and biological indicators, CTCs were classified into epithelial, mesenchymal, and hybrid phenotypes. Specifically, the epithelial CTCs exclusively presented epithelial markers (*EpCAM* and *CK8/18/19*) highlighted by Alexa Fluor 594 (red), while mesenchymal CTCs displayed only the mesenchymal markers (*vimentin* and *twist*) emphasized by Alexa Fluor 488 (green). Hybrid CTCs, possessing both epithelial and mesenchymal attributes, were concurrently stained in both green and red immunofluorescence. All nuclei were tagged with DAPI, manifesting as blue fluorescence.

### Statistical methods

For statistical analysis and graphing, we utilized GraphPad Prism (GraphPad Software, USA) and R language. Importantly, the experiments were repeated three times. To ensure the precision of our results, we first tested the data for normality, homogeneity of variance, and other specific conditions. Depending on these pre-tests, appropriate statistical methods such as the t-test, Wilcoxon rank sum test, ANOVA, and Dunnett’s correction were selected. Furthermore, R was employed to perform t-tests and Chi-squared tests, specifically for analyzing the baseline data post-patient grouping. For visual representation, CTCs paired plots were crafted using the ggplot2 and ggridges packages in R. In our survival analysis, we incorporated variable proportional hazards (PH) hypothetical tests and the Cox proportional hazards regression model, making use of the survminer and survival packages. Other R packages, including car (for VIF), GGally (correlation tests), glmnet (Lasso regression and coefficient screening), rms and e1071 (for nomogram plotting, calibration curves, and SVM classifiers), and maxstat (for determining cutoff values) were also employed. Kaplan–Meier (KM) survival curve analysis was conducted using the survivor, survminer, and ggplot2 packages. Lastly, depending on data characteristics, we chose either Pearson or Spearman tests for correlation analysis. It's worth noting that a P-value less than 0.05 was our threshold for statistical significance, with the respective notations *, **, ***, and **** signifying P-values < 0.05, < 0.01, < 0.001, and < 0.0001.

## Results

### Public database sequencing analysis on CTCs and selection of HUB genes

**Fig. 1:**
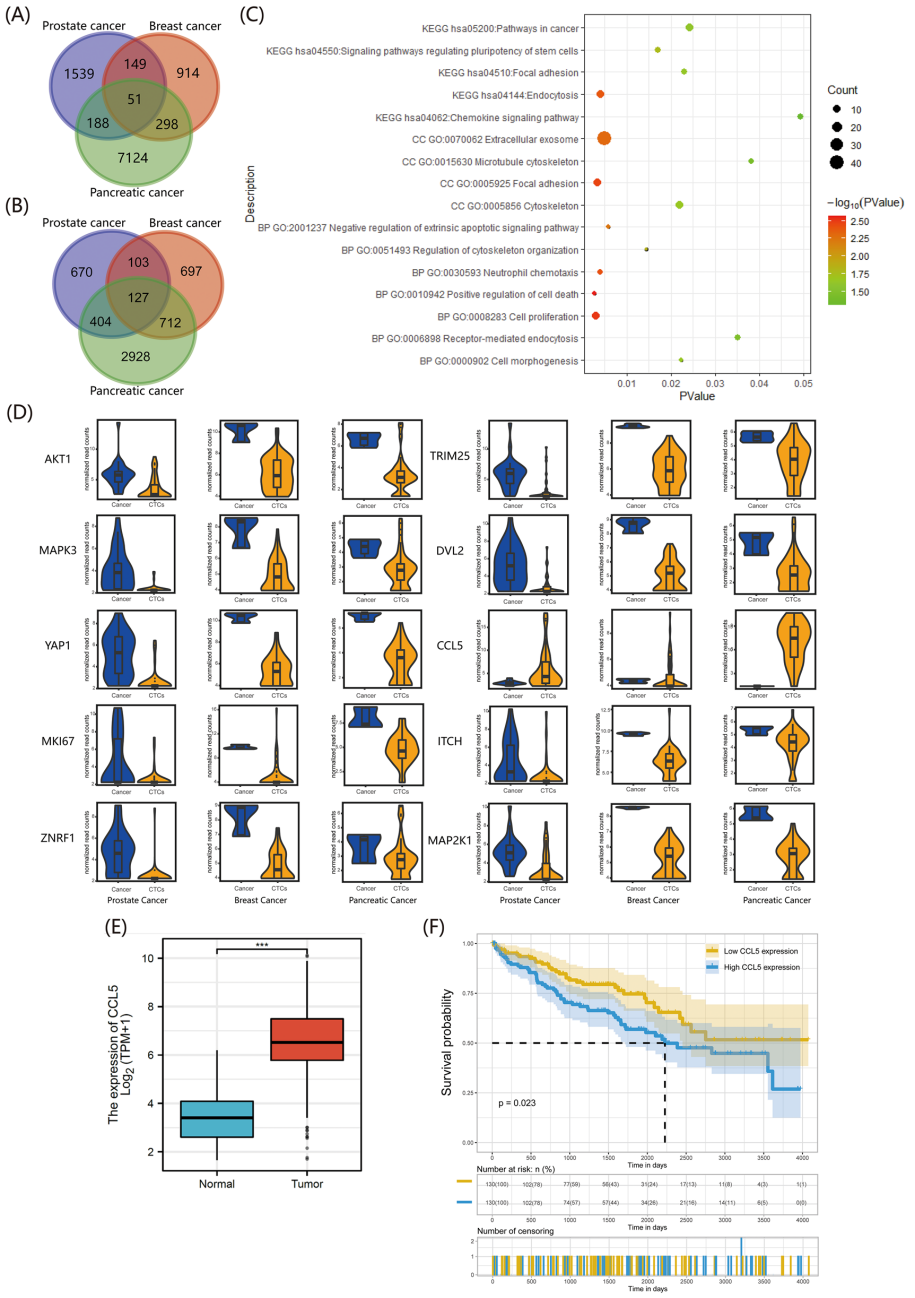
Differential gene analysis between primary tumor cells and circulating tumor cells across various cancers. **A** Venn diagram of upregulated genes in CTCs from prostate, breast and pancreatic cancer. **B** Venn diagram of downregulated genes in CTCs from prostate, breast and pancreatic cancer. **C** KEGG and GO enrichment analysis of differential genes coexisting in prostate, breast and pancreatic cancer. **D** Specific expression of the top 10 HUB genes in primary tumor lesions and CTCs. **E** Expression of CCL5 in renal clear cell carcinoma and adjacent normal tissues in the TCGA database. **F** Kaplan–Meier survival curve analysis demonstrates the effect of CCL5 on the survival probability of patients with renal cancer. The plotting and statistical analyses for bioinformatics were carried out with appropriate packages within R

Since there was no sequencing data related to renal cancer CTCs in the GEO data, in this study we selected sequencing data for CTCs and their corresponding tumor cell lines of prostate cancer, breast cancer, and pancreatic cancer (Table S1). After data standardization, the differential genes were shown in Figure S1, and the results suggested that some genes had significant expression differences between CTCs and tumor cells. Subsequently, with P_adj_ < 0.05 and |logFC|> 1 as screening criteria and using tumor cells as controls, we took the intersection of differentially high or low expressed genes to obtain co-expressed differential genes in the three cancers (Fig. 1 A, B). We also performed enrichment analysis (Fig. 1 C) and found a total of 178 genes with differences in all three cancer CTCs, of which 51 genes were upregulated and 127 genes were downregulated. These co-expressed differential genes mainly focused on cell adhesion, proliferation, and apoptosis, etc. We then visualized the top 10 hub genes (Table S2) (Fig. 1 D). Only CCL5 was highly expressed in CTCs, while the rest were in a low expression state. In the ctcRbase database, we selected different types of cancers (Table S3) and compared the CCL5 expression level in sequencing data (Figure S2). We found that compared to the primary and metastatic sites of the tumor, CCL5 was in a high expression state in CTCs. In the cancer genome atlas (TCGA) database, compared to the adjacent normal tissue, CCL5 was highly expressed in clear cell renal carcinoma tissue (Fig. 1E), and patients with high CCL5 expression in renal cancer were closely related to poor prognosis (Fig. 1F).

### Construction and phenotypic detection of renal cancer circulating tumor cell line 786-O-CTC

**Fig. 2:**
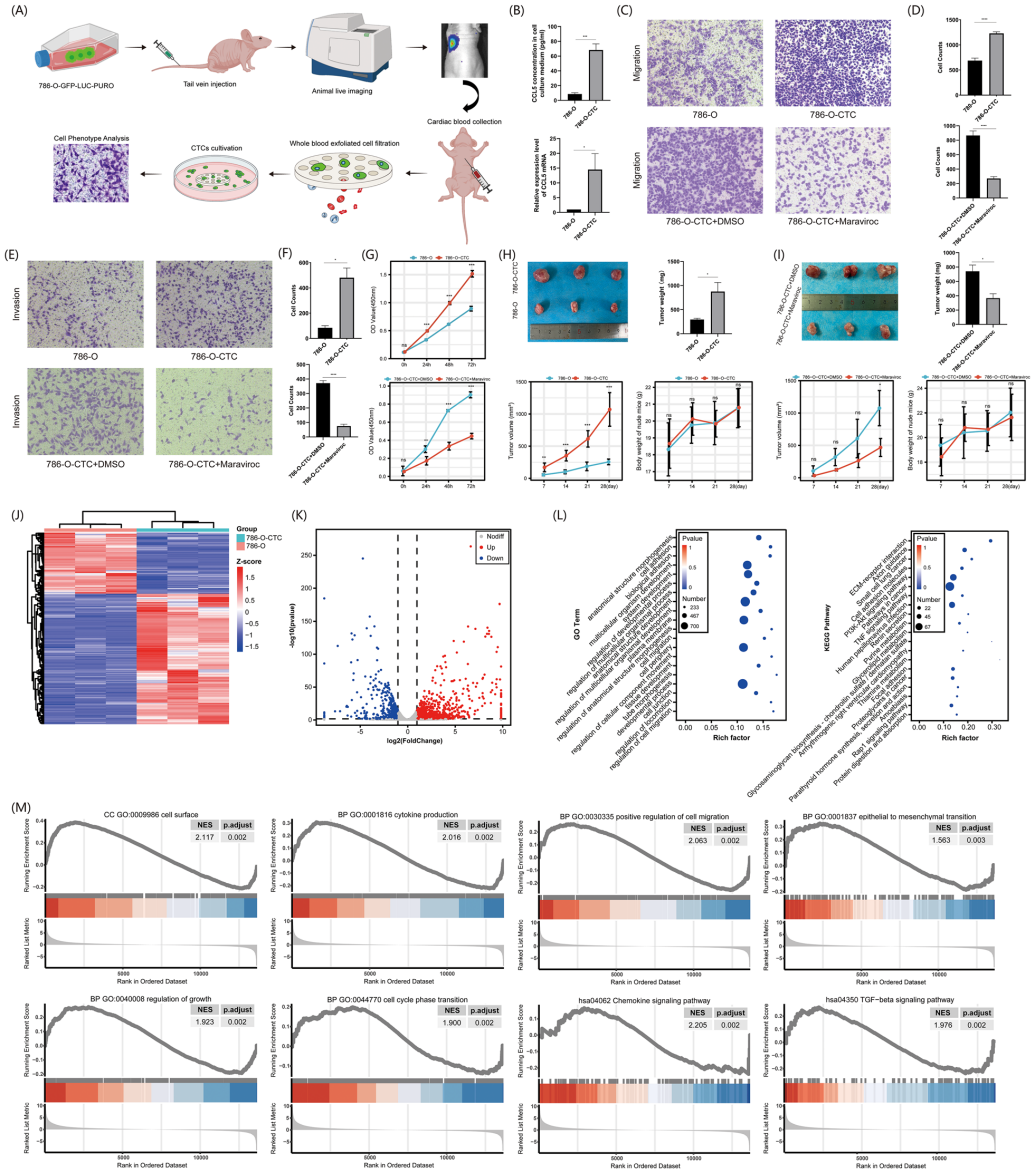
Phenotypic differences and sequencing analysis of 786-O and 786-O-CTC cells. **A** Culture procedure of 786-O-CTC. **B** Above: ELISA analysis of CCL5 protein expression levels in the culture media of 786-O and 786-O-CTC cells. Below: RT-qPCR analysis of CCL5 transcription levels in 786-O and 786-O-CTC cells. **C** Transwell assays assessing changes in cell migration capabilities among 786-O, 786-O-CTC, and 786-O-CTC treated with maraviroc. **D** Above: Quantitative analysis of migrated cell numbers in 786-O-CTC and 786-O. Below: Quantitative analysis of migrated cell numbers in 786-O-CTC under maraviroc intervention. **E** Transwell assays assessing changes in cell invasion capabilities among 786-O, 786-O-CTC, and 786-O-CTC treated with maraviroc. **F** Above: Quantitative analysis of invasive cell numbers in 786-O-CTC and 786-O. Below: Quantitative analysis of invasive cell numbers in 786-O-CTC under maraviroc intervention. **G** Above: Cell proliferation assays for 786-O-CTC and 786-O. Below: Cell proliferation assays for 786-O-CTC under maraviroc intervention. **H** Above Left: Evaluation of tumorigenic potential of 786-O and 786-O-CTC cells in subcutaneous tumor formation experiments in nude mice. Above Right: Quantitative analysis of subcutaneous tumor weights in nude mice from the 786-O and 786-O-CTC groups. Below Left: Quantitative analysis of subcutaneous tumor volumes in nude mice from the 786-O and 786-O-CTC groups. Below Right: Quantitative analysis of the body weights of tumor-bearing mice in the 786-O and 786-O-CTC groups. **I** Above Left: Evaluation of tumorigenic potential of 786-O-CTC and 786-O-CTC under maraviroc intervention in subcutaneous tumor formation experiments in nude mice. Above Right: Quantitative analysis of subcutaneous tumor weights in nude mice from the 786-O-CTC and 786-O-CTC under maraviroc intervention groups. Below Left: Quantitative analysis of subcutaneous tumor volumes in nude mice from the 786-O-CTC and 786-O-CTC under maraviroc intervention groups. Below Right: Quantitative analysis of the body weights of tumor-bearing mice in the 786-O-CTC and 786-O-CTC under maraviroc intervention groups. **J** Heatmap of differential genes between 786-O and 786-O-CTC. **K** Volcano plot of differential genes between 786-O and 786-O-CTC. **L** Left: GO enrichment analysis of differential genes between 786-O and 786-O-CTC. Right: KEGG pathway enrichment analysis of differential genes between 786-O and 786-O-CTC. **M** GSEA analysis of differential genes between 786-O and 786-O-CTC. Experiments involving quantitative analysis were repeated at least three times, with statistical analysis conducted using the t-test

Following the establishment of the renal cancer circulating tumor cell line 786-O-CTC as depicted in Fig. 2A, we examined the expression of CCL5 both at the transcriptional and protein levels (Fig. 2B), revealing an elevated expression of CCL5 in 786-O-CTC. Subsequently, we evaluated the migration and invasion of 786-O-CTC and explored the potential pivotal role of CCL5 in the malignant cellular phenotype by using the CCL5 receptor antagonist maraviroc (Fig. 2C–F). The outcomes indicated that CCL5 augmented the migration and invasion malignant characteristics of 786-O-CTC. Cell proliferation and nude mouse subcutaneous tumorigenesis experiments also indicated that CCL5 can enhance the proliferation and tumorigenicity of 786-O-CTC (Fig. 2 G-I). Then, using P_adj_ < 0.05 and |logFC|> 1 as screening criteria, we performed sequencing analysis on 786-O-CTC and 786-O to obtain their differentially expressed genes (Fig. 2 J, K). Their enrichment analysis indicated that the differentially expressed genes were mainly enriched in cell adhesion, migration, and cytoskeleton reconstruction (Fig. 2L). Further gene set enrichment analysis (GSEA) enrichment analysis corroborated the aforementioned results, highlighting the crucial roles of the EMT and TGF-β pathways (Fig. 2 M).

### CCL5 enhanced the malignant phenotype of CAKI-2 and 786-O cells by promoting EMT

**Fig. 3:**
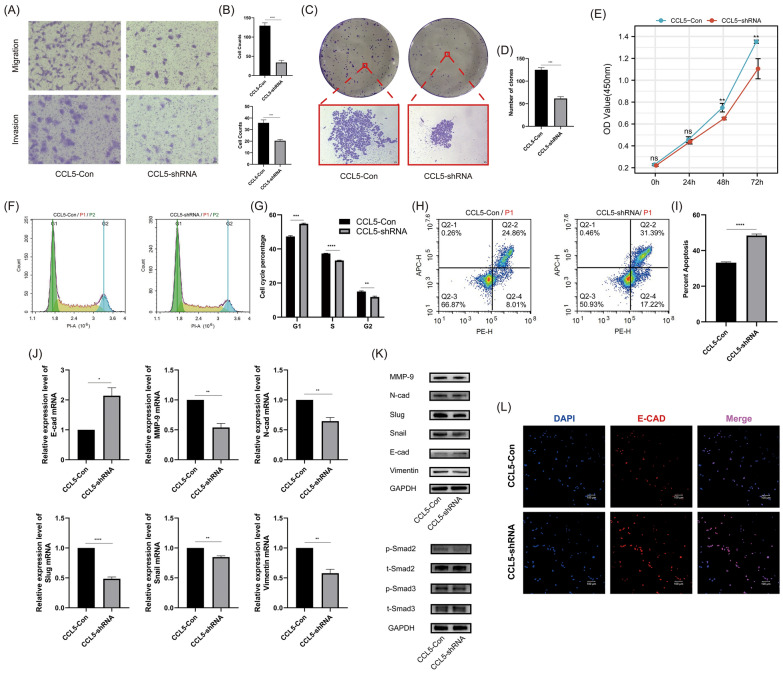
The effect of CCL5 on the phenotype and molecular aspects of CAKI-2 cells. **A** Transwell assays evaluating the effect of CCL5 on the migration and invasion capabilities of CAKI-2 cells. **B** Above: Quantitative analysis of migrated cell numbers in CAKI-2-CCL5-Con and CAKI-2-CCL5-shRNA groups. Below: Quantitative analysis of invasive cell numbers in CAKI-2-CCL5-Con and CAKI-2-CCL5-shRNA groups. **C** Colony formation assays assessing the impact of CCL5 on the cloning efficiency of CAKI-2 cells. **D** Quantitative analysis of colony numbers in CAKI-2-CCL5-Con and CAKI-2-CCL5-shRNA groups. **E** Cell proliferation assays for CAKI-2-CCL5-Con and CAKI-2-CCL5-shRNA groups. **F** Flow cytometry analysis of the effect of CCL5 on the cell cycle of CAKI-2 cells. **G** Quantitative analysis of cell cycle phase distribution changes in CAKI-2-CCL5-Con and CAKI-2-CCL5-shRNA groups. **H** Flow cytometry analysis of CCL5's effect on apoptosis in CAKI-2 cells. **I** Quantitative analysis of apoptosis ratios in CAKI-2-CCL5-Con and CAKI-2-CCL5-shRNA groups. **J** RT-qPCR analysis of the effect of CCL5 on EMT-related markers in CAKI-2 cells. **K** Western blot analysis of the effect of CCL5 on the expression of EMT-related markers and smad2/3 molecules in CAKI-2 cells. **L** Immunofluorescence assay to detect the impact of CCL5 on E-cadherin expression in CAKI-2 cells. Experiments involving quantitative analysis were repeated at least three times, with statistical analysis conducted using the t-test

**Fig. 4:**
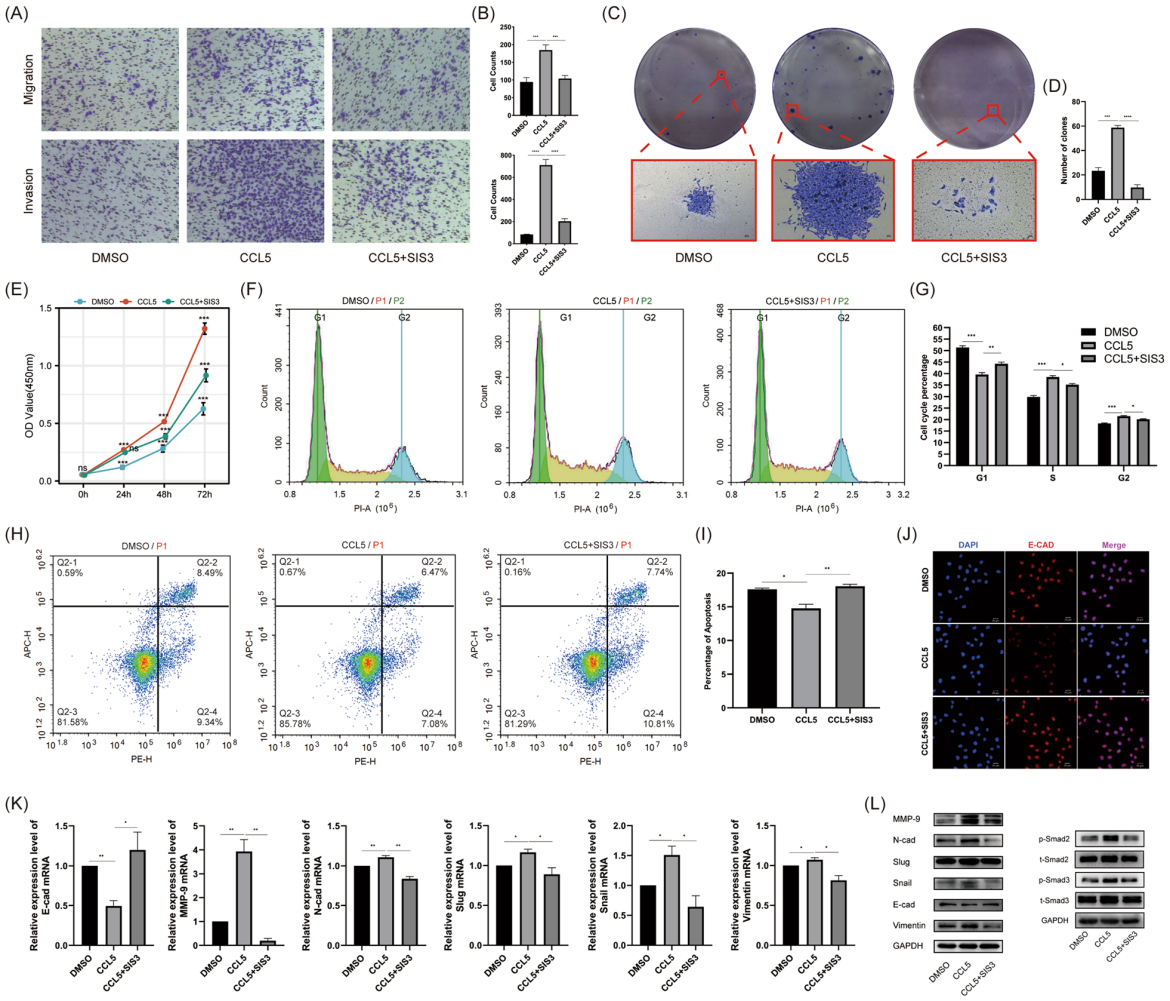
The effect of CCL5 and SIS3 on the phenotype and molecular aspects of 786-O cells. **A** Transwell assays evaluating the effects of CCL5 and SIS3 on the migration and invasion capabilities of 786-O cells. **B** Above: Quantitative analysis of migrated cell numbers in DMSO, CCL5 and CCL5 + SIS3 groups. Below: Quantitative analysis of invasive cell numbers in DMSO, CCL5 and CCL5 + SIS3 groups. **C** Colony formation assays assessing the impact of CCL5 and SIS3 on the cloning efficiency of 786-O cells. **D** Quantitative analysis of colony numbers in DMSO, CCL5 and CCL5 + SIS3 groups. **E** Cell proliferation assays for DMSO, CCL5 and CCL5 + SIS3 groups. **F** Flow cytometry analysis of the effect of CCL5 and SIS3 on the cell cycle of 786-O cells. **G** Quantitative analysis of cell cycle phase distribution changes in DMSO, CCL5 and CCL5 + SIS3 groups. **H** Flow cytometry analysis of the effect of CCL5 and SIS3 on the cell apoptosis of 786-O cells. **I** Quantitative analysis of apoptosis ratios in DMSO, CCL5 and CCL5 + SIS3 groups. **J** Immunofluorescence assay to detect the impact of CCL5 and SIS3 on E-cadherin expression in 786-O cells. **K** RT-qPCR analysis of the effect of CCL5 and SIS3 on EMT-related markers in 786-O cells. **L** Western blot analysis of the effect of CCL5 and SIS3 on the expression of EMT-related markers and smad2/3 molecules in 786-O cells. Experiments involving quantitative analysis were repeated at least three times, with statistical analysis conducted using the t-test

In the common renal carcinoma cell lines 786-O and CAKI-2 that we tested, CCL5 showed higher expression in CAKI-2 and lower in 786-O (Figure S3). Subsequently, based on the baseline expression levels, we knocked down CCL5 in CAKI-2 and verified its knockdown effect (Figure S4). In 786-O, we supplemented with recombinant human CCL5 to mimic overexpression. In CAKI-2 cells, knocking down CCL5 expression can reduce the cells' migration (Fig. 3A, B), invasion (Fig. 3A, B), colony formation (Fig. 3C, D), and proliferation capabilities (Fig. 3E); concurrently, it increased the proportion of cells in the G1 phase and decreased the proportions in the S and G2 phases (Fig. 3F, G). Additionally, it elevated the apoptosis ratio of the cells (Fig. 3 H-I). Transcription (Fig. 3 J) and protein level (Fig. 3 K) assays indicated that knocking down CCL5 in CAKI-2 cells increased the expression of the epithelial marker E-cadherin and reduced the expression of mesenchymal markers such as N-cadherin, MMP9, slug, snail, and vimentin. This occurred alongside a reduction in phosphorylated smad2/3 expression without affecting total smad2/3 protein levels. Additionally, immunofluorescence analysis further supported that knocking down CCL5 enhanced E-cadherin expression levels (Fig. 3L). In 786-O cells, we added SIS3 to inhibit the smad pathway to explore whether the smad pathway played a vital role in the EMT process. As observed in CAKI-2, in 786-O, CCL5 enhanced migration (Fig. 4A, B), invasion (Fig. 4A, B), colony formation (Fig. 4C, D), and proliferation (Fig. 4E), decreased the proportion of cells in the G1 phase and increased the proportions in the S and G2 phases (Fig. 4F, G), and curbs apoptosis (Fig. 4 H, I). However, with the addition of SIS3 intervention, the malignancy boosted by CCL5 saw notable suppression (Fig. 4A–I). Transcription (Fig. 4 K) and protein level (Fig. 4L) assays suggested that CCL5 can inhibit the expression of epithelial markers such as E-cad, promote mesenchymal markers like N-cad, MMP9, slug, snail, vimentin, and simultaneously enhance the protein expression of phosphorylated smad2/3 without changing total smad2/3 protein levels. After treatment with SIS3, the observed alterations in EMT molecules were partially reversed (Fig. 4 K, L). Moreover, immunofluorescence results also supported the inhibitory effect of CCL5 on E-cadherin expression, as well as the reversal of this inhibition by SIS3 (Fig. 4 J). These findings indicated that CCL5 can enhance the malignant phenotype of cells by promoting EMT through the activation of the smad2/3 pathway. When the smad2/3 pathway was inhibited, EMT was also suppressed, and the cell phenotype was rescued.

### CCL5 promoted tumor progression and metastasis by increasing the number of CTCs and facilitating their EMT

**Fig. 5:**
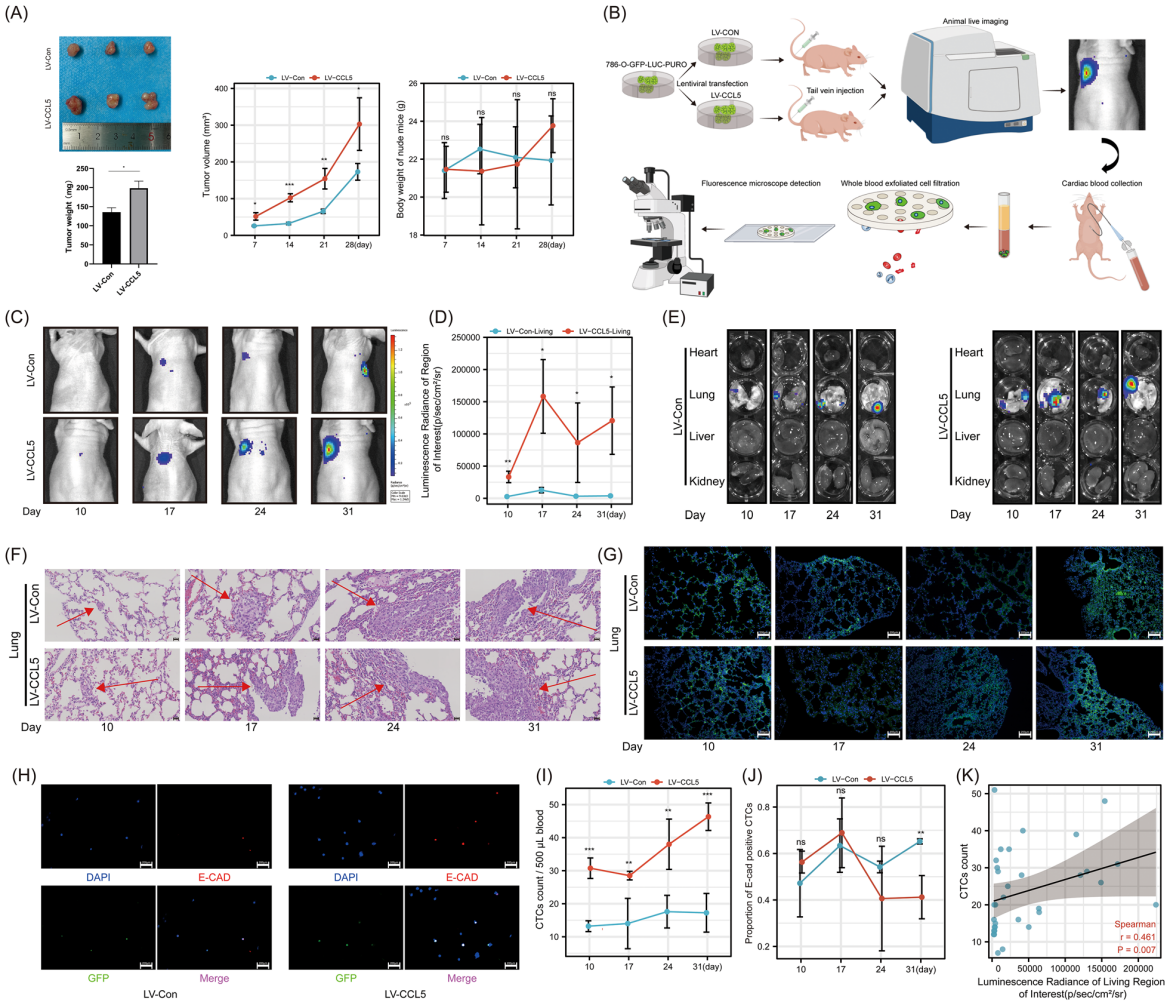
The role of CCL5 in CTCs and tumor metastasis. **A** Above Left: Evaluation of tumorigenic potential of 786-O-LV-CCL5 and 786-O-LV-Con cells in subcutaneous tumor formation experiments in nude mice. Below left: Quantitative analysis of subcutaneous tumor weights in nude mice from the 786-O-LV-CCL5 and 786-O-LV-Con groups. Mid: Quantitative analysis of subcutaneous tumor volumes in nude mice from the 786-O-LV-CCL5 and 786-O-LV-Con groups. Right: Quantitative analysis of the body weights of tumor-bearing mice in the 786-O-LV-CCL5 and 786-O-LV-Con groups. **B** CTCs related animal experiment operation procedure. **C** In vivo imaging evaluating lung metastasis in mice from the 786-O-LV-CCL5 and 786-O-LV-Con groups. **D** Quantitative analysis of the intensity of lung metastasis foci signals in live imaging of mice from the 786-O-LV-CCL5 and 786-O-LV-Con groups. **E** Imaging system assessment of organ metastasis in mice from the 786-O-LV-CCL5 and 786-O-LV-Con groups. **F** HE staining to assess lung metastasis in tumor-bearing mice from the 786-O-LV-CCL5 and 786-O-LV-Con groups. The red arrows indicate the sites of metastasis. **G** Immunofluorescence assessment of lung metastasis in tumor-bearing mice from the 786-O-LV-CCL5 and 786-O-LV-Con groups. (Green represents tumor cells marked with GFP, while blue denotes nuclei stained with DAPI). **H** Immunofluorescence detection and identification of CTCs in the blood of mice from the 786-O-LV-CCL5 and 786-O-LV-Con groups. **I** Quantitative analysis of the number of CTCs in the blood of mice from the 786-O-LV-CCL5 and 786-O-LV-Con groups. **J** Quantitative analysis of the proportion of E-cadherin-positive CTCs in the blood of mice from the 786-O-LV-CCL5 and 786-O-LV-Con groups. **K** Correlation analysis between the number of CTCs and the fluorescence signal intensity of lung metastasis in mice. Experiments involving quantitative analysis were repeated at least three times, with statistical analysis conducted using the t-test, correlation analysis conducted using spearman tests

We overexpressed CCL5 in 786-O cells using a lentivirus and confirmed its reliable overexpression (Figure S5). Subsequent subcutaneous tumorigenesis experiments revealed that CCL5 can enhance the tumorigenicity of tumor cells (Fig. 5A). Concurrently, using the procedure depicted in Fig. 5B, we conducted tumor metastasis experiments in nude mice. In vivo imaging indicated that CCL5 enhanced the lung metastasis associated with CTCs (Fig. 5C, D). Imaging of individual organs, HE, and immunofluorescence results further corroborated the disparities in lung metastasis between the two groups (Fig. 5E–G). Via the capture and staining of CTCs in nude mice (Fig. 5H), we ascertained that CCL5 increased the count of CTCs while diminishing the proportion of E-cad positive CTCs, and there's a positive correlation between CTCs count and the signal intensity of pulmonary metastasis during in vivo imaging (Fig. 5I–K).

### The EMT subtype of CTCs was closely related to the prognosis of renal cancer patients

**Fig. 6:**
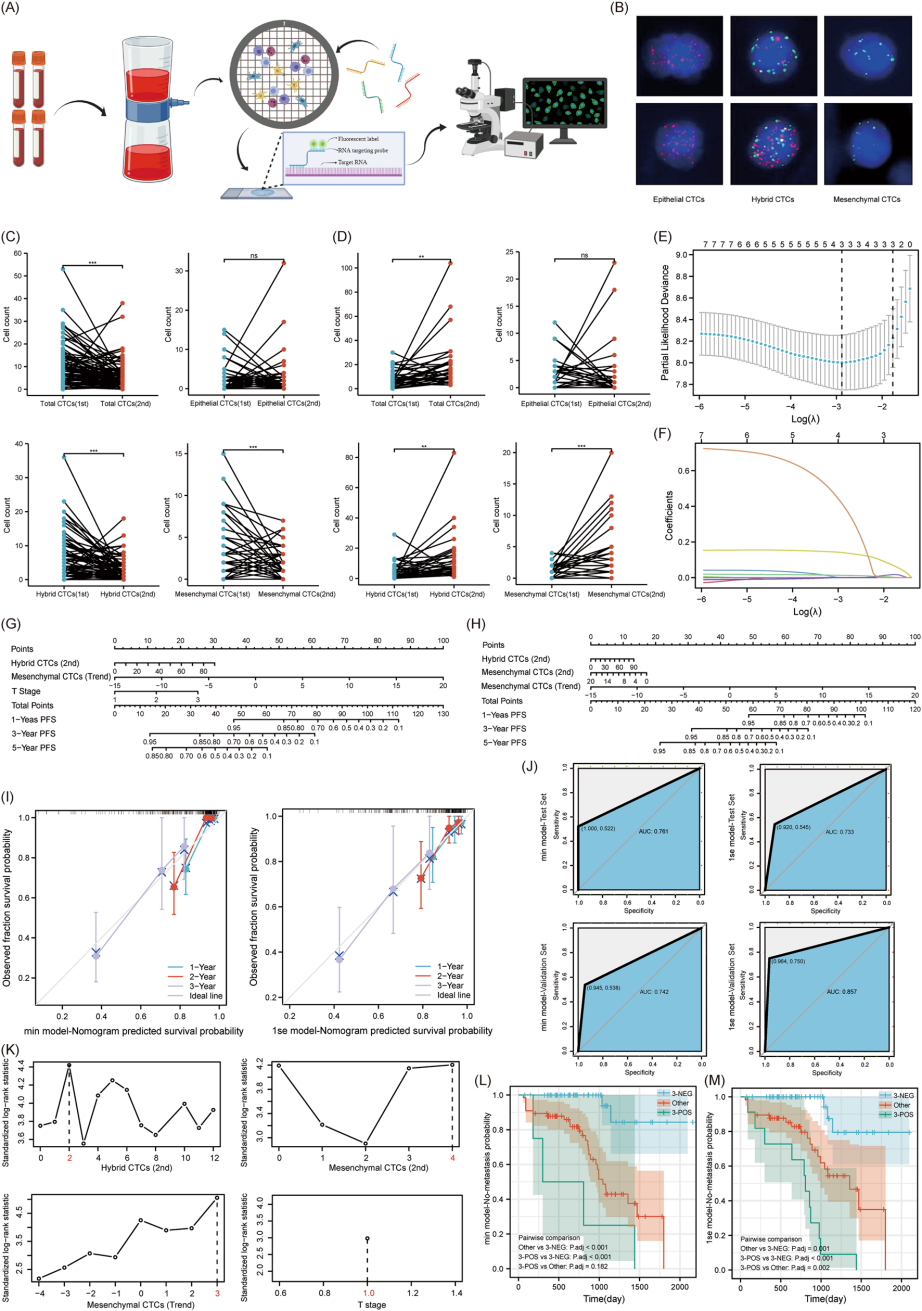
The clinical application value of CTCs in RCC patients. **A** Streamlined process for detecting CTCs in patient blood samples. **B** CTCs of different EMT subtypes in the blood of renal cancer patients (red for epithelial markers, green for mesenchymal markers, blue for DAPI). **C** Variation trend in different EMT subtypes of CTCs for non-metastatic renal cancer patients. **D** Variation trend in different EMT subtypes of CTCs for metastatic renal cancer patients. **E** Prognostic indicators coefficient screening plot. **F** Prognostic indicators variable trajectory plot. **G** Nomogram plot of min model. **H** Nomogram of 1se model. **I** Calibration curve of min and 1se model. **J** Validation of the accuracy of the min model and the 1se model in distinguishing metastatic patients using the SVM algorithm. **K** Determining the best cut-off value for various prognostic factors. **L** Assessing the min model's subgroup differentiation for prognosis with KM survival curves. **M** Assessing the 1se model's subgroup differentiation for prognosis with KM survival curves

Based on the protocol in Figure S6, kidney cancer patients were selected. The baseline information of the enrolled patients was presented in Table 1. This research encompassed 117 RCC patients, with 34 (29.1%) having metastases and 83 (70.9%) without, and the median duration of follow-up being 25 months. Blood samples were taken from enrolled patients, and CTCs categorization and counting were carried out as depicted in Fig. 6A. There were three types of CTCs. Based on biomarkers, they were classified into epithelial CTCs, hybrid CTCs, and mesenchymal CTCs (Fig. 6B). Epithelial CTCs were marked only by epithelial markers (EpCAM and K8/18/19) with Alexa Fluor 594 (red), while mesenchymal CTCs were marked only by mesenchymal markers (vimentin and twist) with Alexa Fluor 488 (green). Hybrid CTCs, displaying both epithelial and mesenchymal markers, were labeled by both green and red fluorescence. Paired analysis between the non-metastatic and metastatic groups indicated that in non-metastatic renal cancer patients, counts of total CTCs, hybrid CTCs, and mesenchymal CTCs trended downwards. However, in metastatic patients, these counts trended upwards (Fig. 6 C, D). After performing the PH test on CTC-related factors, all p-values were greater than 0.05, preliminarily satisfying the prerequisites for the application of the Cox proportional hazards regression model. Initially, we executed a univariate Cox proportional hazards analysis. The findings indicated that the counts of total CTCs, hybrid CTCs, and mesenchymal CTCs from the second examination, along with the trends observed between the two examinations, were significant prognostic indicators (Figure S7). Subsequently, combining significant indicators from the univariate Cox model with tumor T stage, we performed a multivariate Cox proportional hazard model analysis. The results were inconsistent with the univariate model (Figure S8). Diving deeper, we analyzed inter-variable relationships and the Variance Inflation Factor (VIF). Our exploration uncovered that CTC-related variables were strongly interrelated, with all VIFs surpassing 10, highlighting pronounced multicollinearity (Figure S9 and Table S4).

**Table 1:** Association of disease status with clinical pathological variables

	Disease Status		*p*^a^
	No progress (n=83)	Metastasis (n=34)	
	No.	No.	
Age (mean value)	55.73	54.19	0.586^b^
Gender			
Male	51	27	0.061
Female	32	7	
Pathological subtype			
Clear cell carcinoma	70	24	0.089
Others	13	10	
T stage			
T1	73	23	0.018
T2	6	9	
T3	4	2	
Renal score (n=102)			
Low risk	15	2	0.270
Medium risk	41	18	
High risk	17	9	
Differentiation (n=90)			
Low	15	10	0.537
Middle	3	2	
High	43	17	
Surgical approach			
Radical resection	28	17	0.101
Partial resection	55	17	

^a^Chi- square test

^b^Independent sample T test

As a countermeasure, we utilized Lasso regression to mitigate the repercussions of multicollinearity on our prognostic model (Fig. 6E, F). Post-variable compression, we discerned that the prime model encompassed three variables, and we distinguished two alternative models. In the "min" model, three variables were included: the count of hybrid CTCs from the second test, the trend of mesenchymal CTCs, and the T stage. The "1se" model also included three variables: the count of hybrid CTCs and mesenchymal CTCs from the second test and the trend of mesenchymal CTCs. The Nomogram and Calibration plots for the alternative models indicated that the ‘‘1se’’ model more closely mirrored the actual survival rate in the initial triennial period than the ‘‘min’’ model. This insinuates a heightened accuracy in the forecasts of the ‘‘1se’’ model (Fig. 6G–I). Additionally, results from the SVM algorithm showed that the area under the curve (AUC) for the validation set of the ‘‘1se’’ model was 0.857, which was higher than the ‘‘min’’ model’s 0.742, indicating greater accuracy (Fig. 6 J). To streamline the model variables, we transformed them from continuous variables to binary ones. The analysis of cut-off values was shown in Fig. 6 K. For the second test, hybrid CTCs counts ≤ 2 were considered negative, while those > 2 were positive. Mesenchymal CTCs counts ≤ 4 were negative, and those > 4 were positive. For the trend in mesenchymal CTCs, counts ≤ 3 were deemed negative, and those > 3 were positive. For T staging, stage 1 was negative, while stages 2–3 were positive. After simplifying the model, we once again conducted the KM survival curve analysis. In the ‘‘min’’ model, based on the count of hybrid CTCs from the second test, the trend in mesenchymal CTCs, and T staging, kidney cancer patients were classified into three groups: 3-negative, 3-positive, and others. The prognosis was best for the 3-negative group, followed by the others, with the 3-positive group having the worst prognosis. However, the survival difference between the 3-positive group and the others was not statistically significant, with a P-value of 0.182. In the ‘‘1se’’ model, based on the counts of hybrid CTCs and mesenchymal CTCs from the second test, as well as the trend in mesenchymal CTCs, kidney cancer patients were again classified into three groups: 3-negative, 3-positive, and others. The prognosis was best for the 3-negative group, intermediate for the others, and worst for the 3-positive group. There were significant survival differences between any two groups (Fig. 6L, M).

## Discussion

CTCs are pivotal in tumor metastasis, particularly in aggressive malignant tumors prone to hematogenous spread. Simultaneously, CTCs serve as a mirror, reflecting both the dynamic shifts within tumor cells of a single individual and the cellular heterogeneity between different patients. From a clinical standpoint, this knowledge was invaluable in tailoring individualized treatment strategies for tumor patients and assessing their prognosis [9].

Due to the lack of sequencing data related to renal cancer CTCs in databases like GEO, we took the intersection of differential genes in CTCs across various cancers and proceeded with analysis (Figure S1). This method is reminiscent of the pan-cancer analysis previously employed to delve into the roles of oncogenes across diverse cancers [10]. In the end, we identified 178 co-expressed genes that showed variations in prostate cancer, breast cancer, and pancreatic cancer. Concurrently, enrichment analysis indicated that the primary functions of these genes centered on intercellular adhesion, skeletal reconstruction, proliferation, apoptosis, etc. (Fig. 1). During the formation of CTCs, when tumor cells detach from the primary lesion and enter the bloodstream, the expression of their adhesion molecules decreases. This accelerates the degradation of the extracellular matrix, thus promoting cell detachment and metastasis [11]. Among the genes that facilitate this invasive phenotype, CCL5 stood out as the only one among the TOP 10 HUB genes that's overexpressed in CTCs. CCL5 is part of the C–C chemokine family, which plays a pivotal role in tumor progression and can propel tumor development in a multitude of ways[12–14]. CCL5 can alter the polarization state of the cytoskeleton by activating a series of downstream effector molecules, thus facilitating the remodeling and migration of components integral to the extracellular matrix [15]. The analysis from the ctcRbase database suggested that CCL5 expression in CTCs was consistently high across different cancers (Figure S2). When CTCs are entrapped by capillaries, the CCL5 they secrete can induce macrophage recruitment to the vessel wall surrounding the CTCs. Subsequently, these macrophages release VEGF, enhancing the permeability of vascular endothelial cells and making it more likely for CTCs to escape from blood vessels [16]. In renal cancer, CCL5 was highly expressed in tumor tissues and correlated with a poor prognosis (Fig. 1). Thus, we proposed that CCL5 might be overexpressed in renal cancer CTCs and might enhance the malignant phenotype of the cells.

We established the renal cancer circulating tumor cell line 786-O-CTC and investigated the effect of CCL5 on its invasive phenotype. The results suggested that CCL5 can enhance the migration, invasion, proliferation, and subcutaneous tumorigenic ability of 786-O-CTC. Sequencing results indicated that genes differentially expressed between 786-O-CTC and 786-O mainly enriched in cellular adhesion and migration. Additionally, GSEA enrichment analysis suggested that EMT and the TGF-β pathway played crucial roles in this process (Fig. 2). We hypothesized that CCL5 promoted the EMT process in renal cancer cells, which in turn impacted CTCs and ultimately led to a poor prognosis. Therefore, we further validated the role of CCL5 in EMT using renal cancer cells. In CAKI-2 cells, CCL5 enhanced capabilities such as invasion, clonal formation, promoted cell proliferation, and inhibited apoptosis. This invasive phenotype was primarily acquired through EMT, with smad2/3 playing a pivotal role (Fig. 3). We then revalidated the promoting effect of CCL5 on EMT in 786-O cells at both cellular and molecular levels. We also introduced the smad2/3 inhibitor SIS3 as an intervention group to further confirm the importance of the smad pathway (Fig. 4). Similar to our results, in prostate cancer, CCL5 can enhance cell migration and invasion by promoting EMT [17]. In colorectal cancer, inhibiting the phosphorylation of smad2/3 can further suppress EMT, ultimately curbing malignant cell phenotypes like migration and invasion [18]. To further validate the tumorigenic role of CCL5 in vivo, we first conducted subcutaneous tumorigenesis experiments, preliminarily confirming that CCL5 can enhance tumorigenic ability (Fig. 5). This was consistent with Wang's study, where in an osteosarcoma xenograft tumor model, direct knockdown of CCL5 in tumor cells also significantly inhibited tumor growth and angiogenesis [19]. Moreover, through the metastatic tumor model, we found that CCL5 can increase the number of renal cancer CTCs, promote EMT, and enhance lung metastasis (Fig. 5). In line with our findings, direct or indirect regulation of CCL5 had shown significant effects on mouse xenograft tumor size and metastasis. For instance, miR-147a can suppress CCL5 expression in non-small cell lung cancer, and regulating CCL5 indirectly using this method had been found to significantly inhibit mouse xenograft tumor growth and metastasis [20]. Sun and his team conducted single-cell sequencing of liver cancer CTCs and discovered that CCL5, regulated by the p38-MAX signaling pathway, was overexpressed in CTCs. This led to the further recruitment of regulatory T cells, promoting immune escape and metastatic seeding of CTCs [21]. Hence, we speculated that CTCs with different EMT phenotypes might have varying prognostic implications in renal cancer patients.

In our study, we incorporated 117 kidney cancer patients (Figure S6), with 34 (29.1%) experiencing metastasis and 83 (70.9%) without any metastasis. The detected CTCs were classified into three types: epithelial, hybrid, and mesenchymal types (Fig. 6). In the data we observed, the counts for total, hybrid, and mesenchymal CTCs showed an upward trend in the metastatic group. Contrarily, these counts trended downwards in the nonmetastatic group (Fig. 6). This indicated that mesenchymal CTCs might have a more pronounced role in tumor metastasis compared to epithelial CTCs. In our prior studies, we observed that an elevated ratio of mesenchymal CTCs typically indicates an adverse prognosis [7, 8]. While CTCs provide valuable insights, some studies have indicated that results from a single CTC detection can be uncertain. Specifically, in liver cancer, individual CTC typing and counting outcomes do not necessarily correlate with the clinical stage or tumor recurrence in patients [22]. Thus, in this study, we tested CTCs in each renal cancer patient at least twice and conducted both univariate and multivariate Cox hazard ratio analyses on the classification, count, and trend changes of CTCs (Figure S7-8). After identifying that collinearity and correlation might affect the analysis results (Figure S9, Table S4), we carried out a Lasso analysis on CTC-related indicators, including tumor T-stage. By reducing the impact of correlation and collinearity, we selected the prognosis model. Through the use of column plots, calibration curves, and support vector machine (SVM) algorithm comparisons, we determined that the “1se” model containing single counts of hybrid and mesenchymal CTCs, as well as the trend change of mesenchymal CTCs, was the optimal model (Fig. 6). In our analysis, the three variables of the “1se” model were intimately tied to the count of mesenchymal CTCs. This finding aligned with our previous research, suggesting that a heightened presence of mesenchymal CTCs in RCC patients might correlate with a poorer prognosis [23]. This phenomenon can be largely attributed to the EMT process. This process led to the loss of tumor cell polarity, diminished intercellular adhesion, and alterations in epithelial and mesenchymal markers in tumor cells. Consequently, tumor cells undergo morphological changes, becoming more invasive and easier to detach from the primary tumor [24, 25]. Other research corroborated that, compared to epithelial CTCs, mesenchymal CTCs resisted the destructive effects of blood flow shear force more effectively [26, 27]. When examining CTCs in other cancer types, our conclusions resonated with established findings. For instance, Chen's research pinpointed that hybrid and mesenchymal CTC proportions in patients with hepatocellular carcinoma (HCC) were influenced by factors such as age, Barcelona Clinic Liver Cancer Stage, metastasis, and alpha-fetoprotein levels. It was observed that patients with recurrent HCC typically exhibited higher CTC counts and a rising percentage of hybrid and mesenchymal CTCs [28]. Echoing this, Yu et al. uncovered a significant link between mesenchymal CTCs and disease advancement in a study of long-term serial CTCs from 11 breast cancer patients [29]. Subsequently, we applied a cutoff value to binarize the prognostic factors and validated the accuracy and comprehensiveness of the “1se” model using the KM survival curve (Fig. 6). The model was capable of capturing instances where the CTC counts were high yet with vague changing trends, and situations where the CTC counts were low but demonstrated evident trends. Through an integrated analysis of the model, we can accurately determine the prognosis for kidney cancer patients, enabling timely interventions to enhance their prognosis.

## Conclusion

In our research, utilizing bioinformatics analysis, we suggested that CCL5 advances the malignancy of kidney cancer CTCs. Subsequent in vivo and in vitro experiments and clinical data investigations revealed that CCL5 can enhance the metastatic ability of renal cancer CTCs through smad2/3-induced EMT. In patients with renal cancer, mesenchymal CTC-related indicators can stratify the risk of metastasis, accurately predict prognosis, and facilitate early intervention.

### Supplementary Information

Supplementary material file1.

## Data Availability

The data that support the findings of this study are available from the corresponding author upon reasonable request.

## Abbreviations

CCL5: C–C motif chemokine ligand 5
CTCs: Circulating tumor cells
EMT: Epithelial mesenchymal transition
RCC: Renal cell carcinoma
