# Supplementary material for: CCL5 promotes the epithelial-mesenchymal transition of circulating tumor cells in renal cancer
Source: J Transl Med. 2024 Sep 3;22:817. doi: 10.1186/s12967-024-05297-2 (PMC11370314; doi:10.1186/s12967-024-05297-2)
Supplement: Supplementary file 1 — Supplementary material file1. [file 12967_2024_5297_MOESM1_ESM.docx]

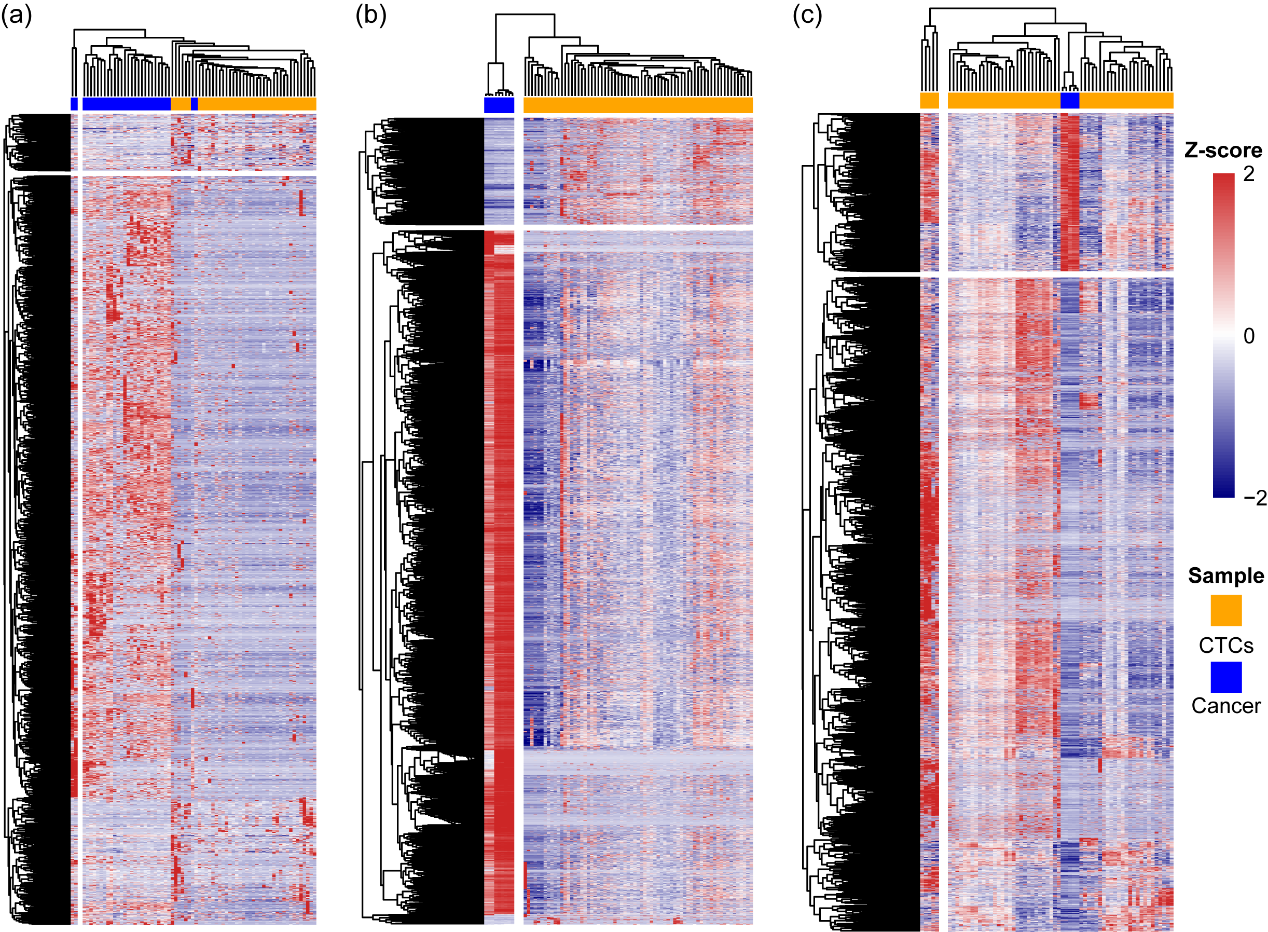
Figure S1

Heatmap of differential genes between primary tumor cells and circulating tumor cells. (a) Heatmap of differential genes in prostate cancer. (b) Heatmap of differential genes in breast cancer. (c) Heatmap of differential genes in pancreatic cancer.


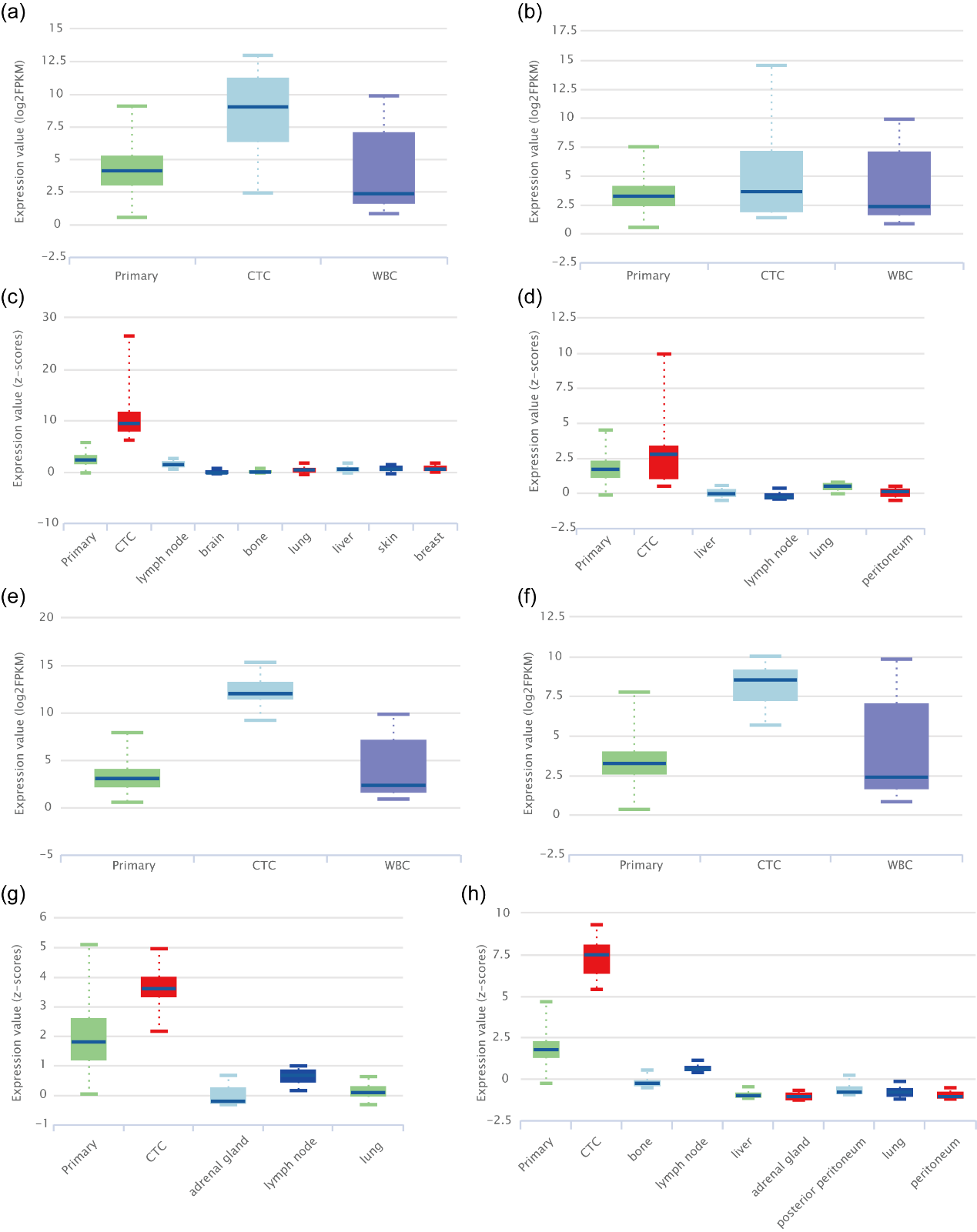
Figure S2

CCL5 expression in different regions among diverse cancer patients. (a) CCL5 expression across different regions in breast cancer patients. (b) CCL5 expression across different regions in colorectal cancer patients. (c) CCL5 expression in different metastatic sites of breast cancer patients. (d) CCL5 expression in different metastatic sites of colorectal cancer patients. (e) CCL5 expression across different regions in liver cancer patients. (f) CCL5 expression across different regions in prostate cancer patients. (g) CCL5 expression in different metastatic sites of liver cancer patients. (h) CCL5 expression in different metastatic sites of prostate cancer patients.


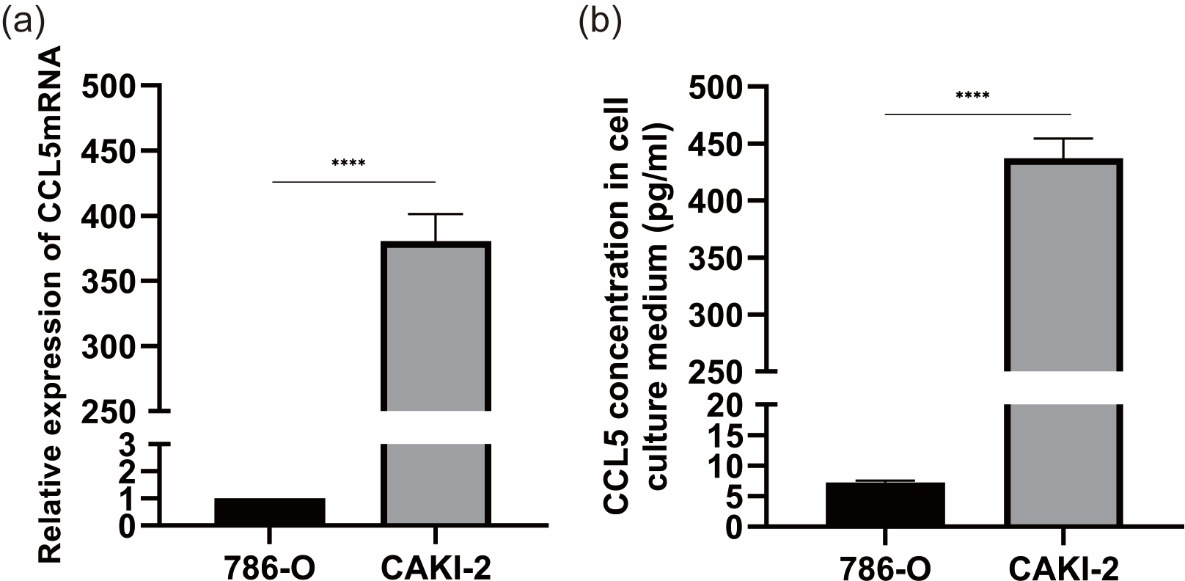
Figure S3

Baseline expression level assessment of CCL5 in 786-O and CAKI-2. (a) RT-qPCR analysis of CCL5 transcription. (b) ELISA assessment of CCL5 protein expression in the culture medium.


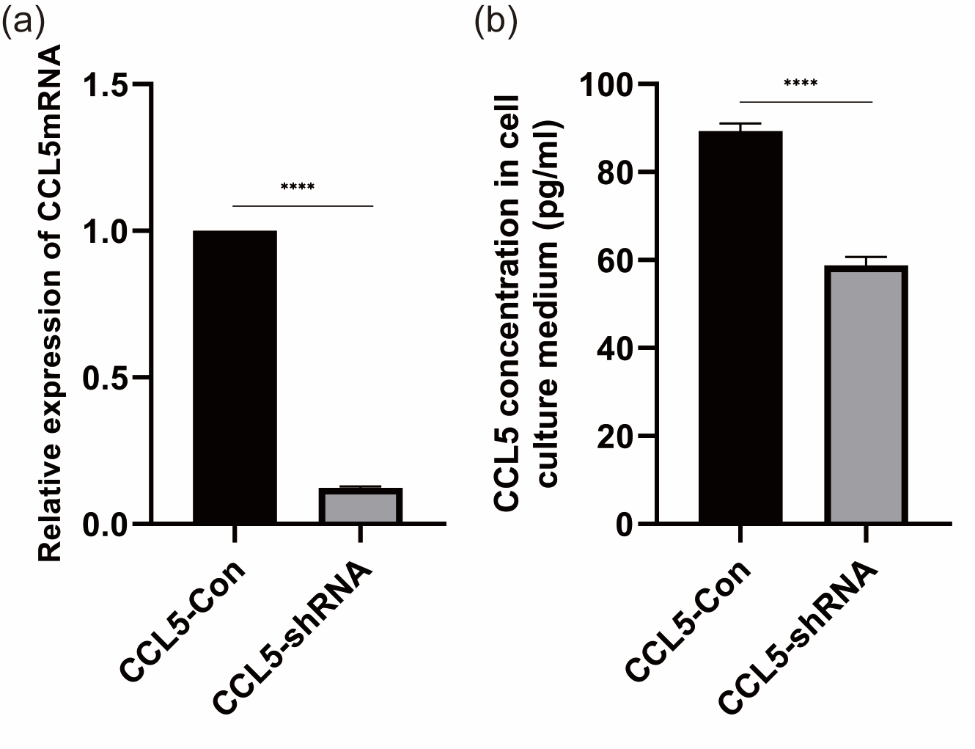


Figure S4

Assessment of CCL5 expression in CAKI-2 cells post-gene manipulation. (a) RT-qPCR analysis of CCL5 transcription. (b) ELISA assessment of CCL5 protein expression in the culture medium.


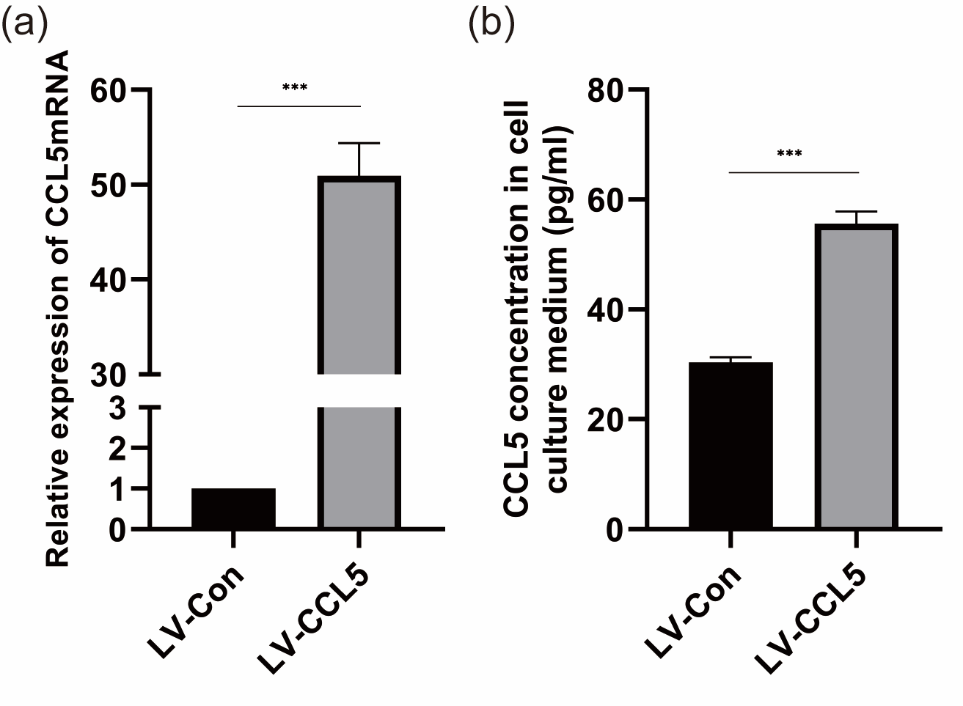


Figure S5

Assessment of CCL5 expression in 786-O cells post-gene manipulation. (a) RT-qPCR analysis of CCL5 transcription. (b) ELISA assessment of CCL5 protein expression in the culture medium.


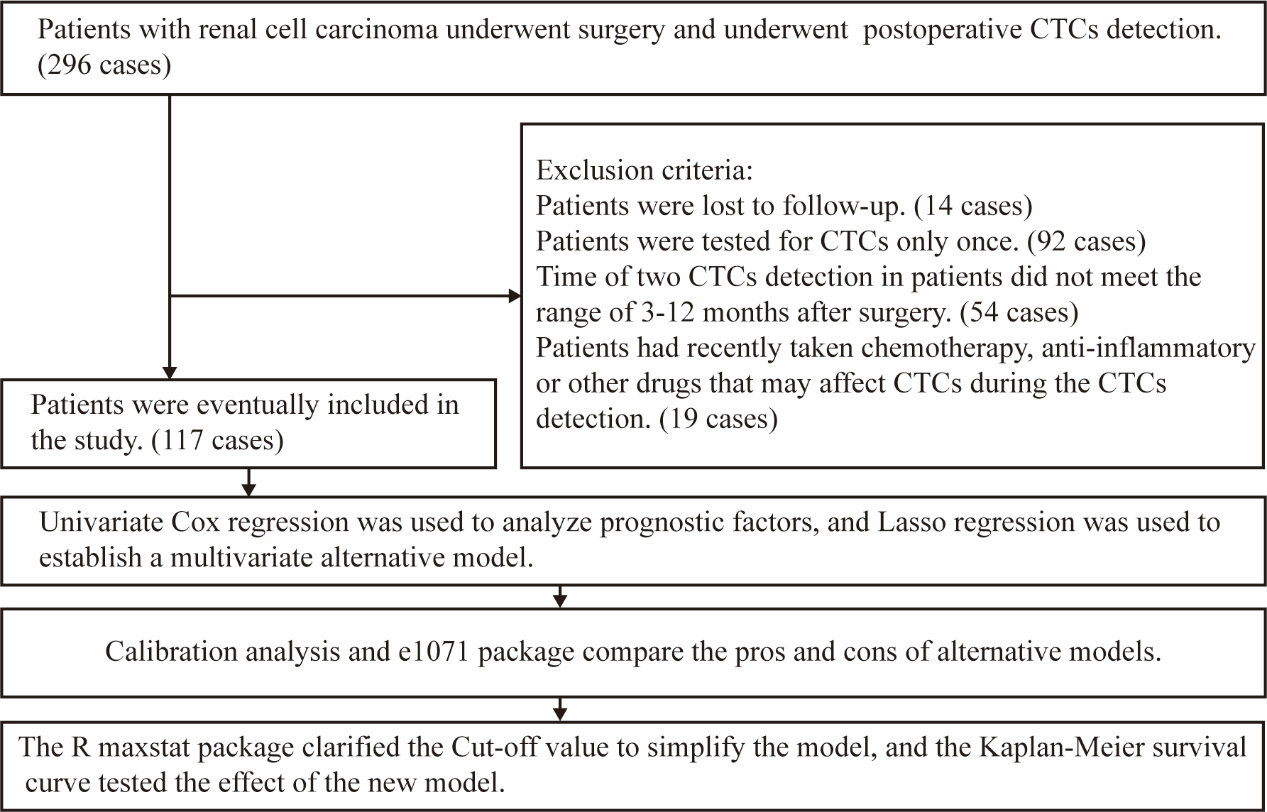
Figure S6

Inclusion and exclusion criteria and data analysis process for renal cancer patients.


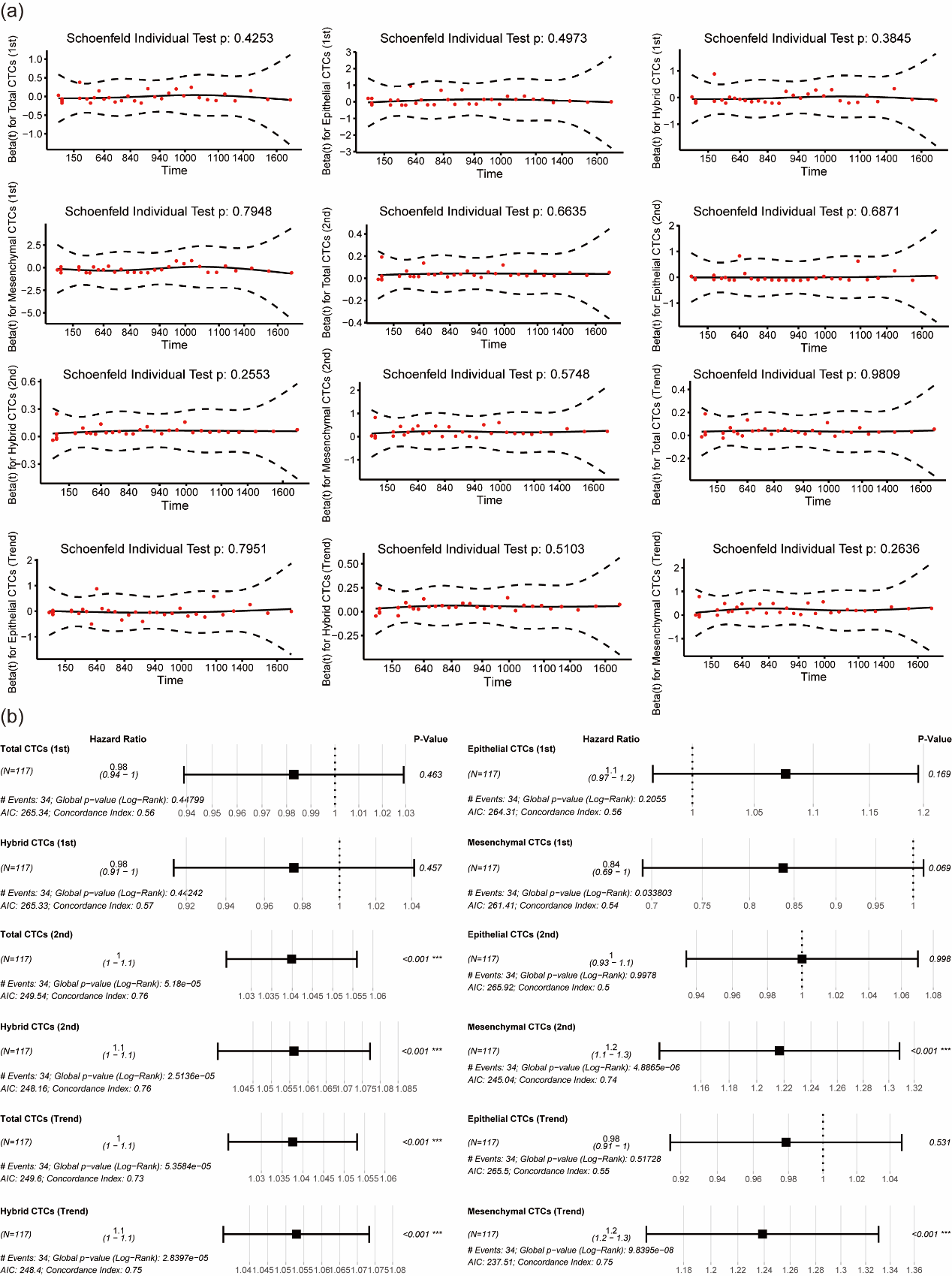
Figure S7

(a) Analysis of variable proportional hazards (PH) hypothetical based on CTCs typing and counting proportions. (b) Univariate Cox proportional hazards regression model based on CTCs typing and counting.


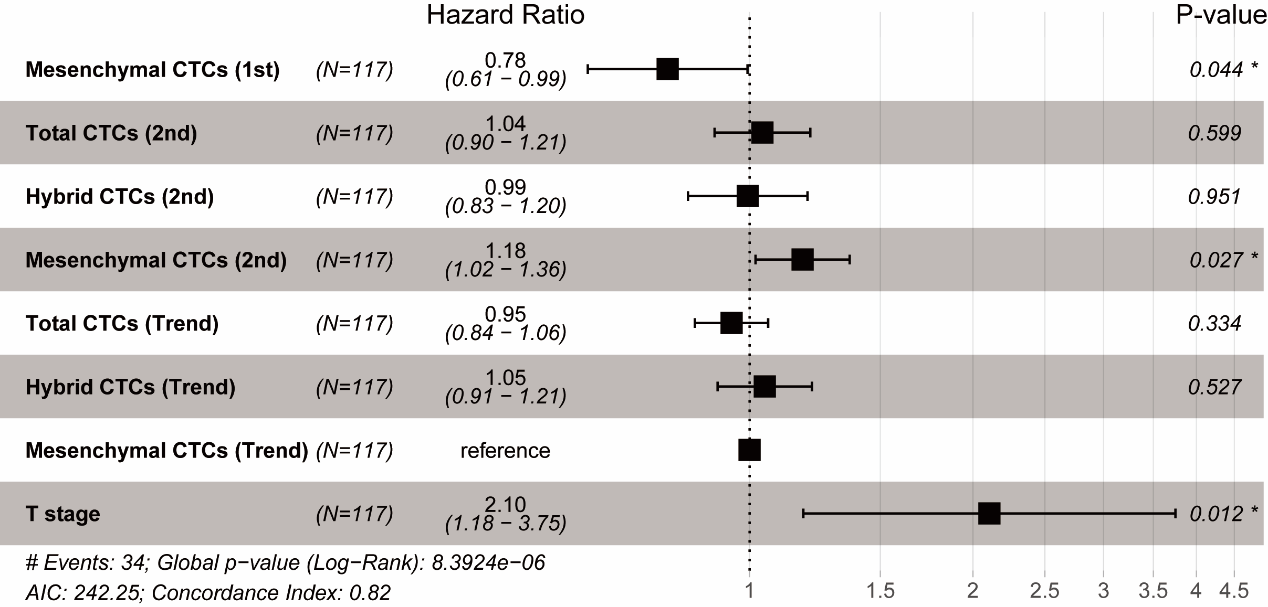


Figure S8

Multivariate Cox proportional hazards regression model based on CTCs typing and counting.


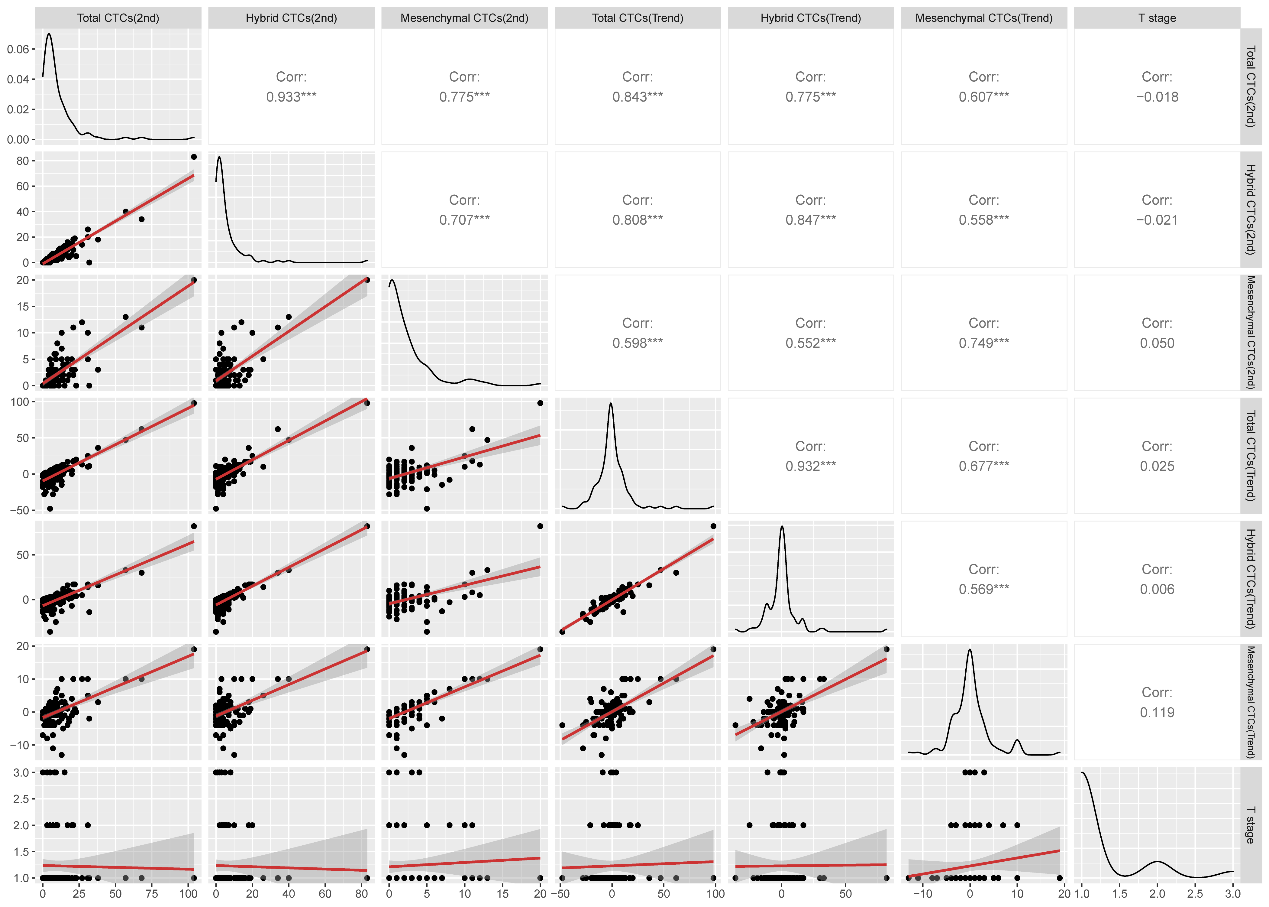
Figure S9

Variable correlation analysis.

Table S1. Basic information about the data set included in the analysis

| Cancer | Dataset ID | CTCs | Cancer cell lines | Total number |  |
| --- | --- | --- | --- | --- | --- |
| Prostate cancer | GSE67980 | 41 | 30 | 71 | |
| Breast cancer | GSE111065 | 69 | 0 | 78 | |
|  | GSE74981 | 0 | 9 |  |  |
| Pancreatic cancer | GSE144561 | 60 | 0 | 65 | |
|  | GSE82104 | 0 | 2 |  |  |
|  | GSE85610 | 0 | 3 |  |  |

|  | Table S2 Top 10 HUB genes with high radiality |  |
| --- | --- | --- |
| Gene symbol | Gene description | Radiality |
| AKT1 | AKT serine/threonine kinase 1 | 5.19 |
| MAPK3 | Mitogen-activated protein kinase 3 | 4.99 |
| YAP1 | Yes1 associated transcriptional regulator | 4.85 |
| MKI67 | Marker of proliferation Ki-67 | 4.83 |
| ZNRF1 | Zinc and ring finger 1 | 4.78 |
| TRIM25 | Tripartite motif containing 25 | 4.76 |
| DVL2 | Dishevelled segment polarity protein 2 | 4.73 |
| CCL5 | C-C motif chemokine ligand 5 | 4.71 |
| ITCH | Itchy E3 ubiquitin protein ligase | 4.69 |
| MAP2K1 | Mitogen-activated protein kinase kinase 1 | 4.67 |

| Table S3 Details of samples selected from the ctcRbase database | | | | |
| --- | --- | --- | --- | --- |
| Database ID | Category | Cancer Type | CTC Extract Method | Dataset |
| BRCA00640 | mRNA | breast cancer | Cluster-Chip | GSE67939 |
| COAD00910 | mRNA | colorectal cancer | microfiltration immunofluorescence | GSE74369 |
| LIHC00011 | mRNA | liver cancer | CTC-iChip immunofluorescence | GSE117623 |
| PRAD00190 | mRNA | prostate cancer | CTC-iChip | GSE104209 |

| Table S4 Variance Inflation Factor (VIF) statistics table | | |
| --- | --- | --- |
| Category | Variable | VIF |
| Second examination | Total CTCs count | 98.7 |
|  | Hybrid CTCs count | 90.4 |
|  | Mesenchymal CTCs count | 14.8 |
| Trend of change | Total CTCs count | 61.9 |
|  | Hybrid CTCs count | 62.1 |
|  | Mesenchymal CTCs count | 12.4 |
| / | T stage | 1.2 |

Table S5 Nucleic acid probe sequence

| Gene | Sequence (5'→3') |
| --- | --- |
| EpCAM | TGGTGCTCGTTGATGAGTCAAGCCAGCTTTGAGCAAATGA |
|  | AAAGCCCATCATTGTTCTGGCTCTCATCGCAGTCAGGATC |
|  | TCCTTGTCTGTTCTTCTGACCTCAGAGCAGGTTATTTCAG |
|  |  |
| CK8 | CGTACCTTGTCTATGAAGGAACTTGGTCTCCAGCATCTTG |
|  | CCTAAGGTTGTTGATGTAGCCTGAGGAAGTTGATCTCGTC |
|  | CAGATGTGTCCGAGATCTGGTGACCTCAGCAATGATGCTG |
|  |  |
| CK18 | AGAAAGGACAGGACTCAGGCGAGTGGTGAAGCTCATGCTG |
|  | TCAGGTCCTCGATGATCTTGCAATCTGCAGAACGATGCGG |
|  | AAGTCATCAGCAGCAAGACGCTGCAGTCGTGTGATATTGG |
|  |  |
| CK19 | CTGTAGGAAGTCATGGCGAGAAGTCATCTGCAGCCAGACG |
|  | CTGTTCCGTCTCAAACTTGGTTCTTCTTCAGGTAGGCCAG |
|  | CTCAGCGTACTGATTTCCTCGTGAACCAGGCTTCAGCATC |
|  |  |
| Vimentin | GAGCGAGAGTGGCAGAGGACCTTTGTCGTTGGTTAGCTGG |
|  | CATATTGCTGACGTACGTCAGAGCGCCCCTAAGTTTTTAA |
|  | AAGATTGCAGGGTGTTTTCGGGCCAATAGTGTCTTGGTAG |
|  |  |
| Twist | ACAATGACATCTAGGTCTCCCTGGTAGAGGAAGTCGATGT |
|  | CAACTGTTCAGACTTCTATCCCTCTTGAGAATGCATGCAT |
|  | TTTCAGTGGGCTGATTGGCACTTACCATGGGTCCTCAATAA |
|  |  |
| CD45 | TCGCAATTCTTATGCGACTCTGTCATGGAGACAGTCATGT |
|  | GTATTTCCAGCTTCAACTTCCCATCAATATAGCTGGCATT |
|  | TTGTGCAGCAATGTATTTCCTACTTGAACCATCAGGCATC |
